# Cost-Effectiveness Analysis of Contemporary Advanced Prostate Cancer Treatment Sequences †

**DOI:** 10.3390/curroncol32040240

**Published:** 2025-04-20

**Authors:** Valentyn Litvin, Armen G. Aprikian, Alice Dragomir

**Affiliations:** 1 Faculty of Pharmacy, University of Montréal, Montréal, QC H3T 1J4, Canada; valentyn.litvin@umontreal.ca; 2 Division of Urology, McGill University, Montréal, QC H4A 3J1, Canada; armen.aprikian.med@ssss.gouv.qc.ca

**Keywords:** prostate cancer, androgen-receptor pathway inhibitor, cost-effectiveness, treatment sequence

## Abstract

There has been a proliferation of novel treatments for the management of advanced prostate cancer (PCa), including androgen receptor pathway inhibitors (ARPI). Although there are health economic analyses of novel PCa treatments, such as ARPIs for specific health states, there is a lack of sequential analyses. Our paper aims to fill this gap. We developed a Monte Carlo Markov model to simulate the management of advanced PCa to end-of-life. We modeled patients who begin in metastatic and nonmetastatic castration-sensitive PCa (mCSPC and nmCSPC), with risk stratification for mCSPC, progressing to metastatic castration-resistant PCa (mCRPC). Using current guidelines and recent literature, we simulated admissible treatment sequences over these states along a 15-year horizon. We report the best treatment sequences in terms of efficacy and cost-effectiveness. We find that the most cost-effective use of ARPIs is early in advanced PCa for a cost-effectiveness threshold (CET) of CAD 100K per QALY. For a CET of CAD 50K per QALY, early ARPI use is most cost-effective in mCSPC-starting patients but not nmCSPC-starting. We conclude that the most cost-effective way to use ARPIs is when patients first enter advanced PCa. The most cost-effective ARPI at current Canadian prices is abiraterone, mostly due to abiraterone’s lower price level.

## 1. Introduction

Prostate cancer (PCa) is a common cancer with a large health impact, accounting for over 375,000 deaths worldwide annually [1]. In an effort to alleviate this health burden, there is continuous innovation to improve upon the long-standing core of PCa management, androgen deprivation therapy (ADT). There has been a recent expansion of available treatments to augment ADT, including the addition of promising androgen receptor pathway inhibitors (ARPI) since the 2010s, namely abiraterone, apalutamide, darolutamide, and enzalutamide.

These drugs have been shown to improve patient outcomes for a wide range of health states, including high-risk nonmetastatic castration-sensitive PCa (nmCSPC) [2], metastatic castration-sensitive PCa (mCSPC) [3,4,5,6,7,8], metastatic castration-resistant PCa (mCRPC) [9,10,11], and nonmetastatic castration-resistant PCa (nmCRPC) [12,13,14]. Currently, ARPIs are approved and recommended for mCSPC [15,16], mCRPC [15,17], and nmCRPC [15,17] patients.

Despite the widespread use of ARPIs and the associated research on their efficacy and cost-effectiveness, there is still an open question about the cost-effectiveness of treatment sequences with different ARPI timing. Our goal was to fill this gap by finding the most cost-effective advanced PCa treatment sequences, with a focus on the Canadian health-economic context.

In our analysis, patients start in either nmCSPC or mCSPC and progress onto nmCRPC and/or mCRPC and then death. Reflecting available outcome data, simulated patients are identical and not stratified by other covariates such as age. We applied clinical guidelines to select allowable treatment sequences and aimed our health–economic analysis toward a Canadian public payer perspective. Using a Monte Carlo Markov model, we simulated population-level patient outcomes over a 15-year horizon for all treatment sequences. We used these results to compare sequences in terms of efficacy and cost-effectiveness.

## 2. Materials and Methods

### 2.1. Study Design Overview

We developed a decision-analytic model to compare different sequences of PCa treatments, incorporating a Monte Carlo Markov model to simulate patient outcomes. The approved treatment sequences are taken from National Comprehensive Cancer Network (NCCN) and Canadian Urological Association (CUA) guidelines [15,16,17], and the costs aim to reflect the perspective of a Canadian public payer. We report our results in quality-adjusted life-years (QALY) and life-years (LY). We use cost-effectiveness thresholds (CETs) of CAD 50K per QALY and CAD 100K per QALY and LY. We take these threshold values from recent health technology assessments in the Canadian oncological context conducted by Canada’s Drug Agency (CDA), formerly known as CADTH [18].

**Figure 1:**
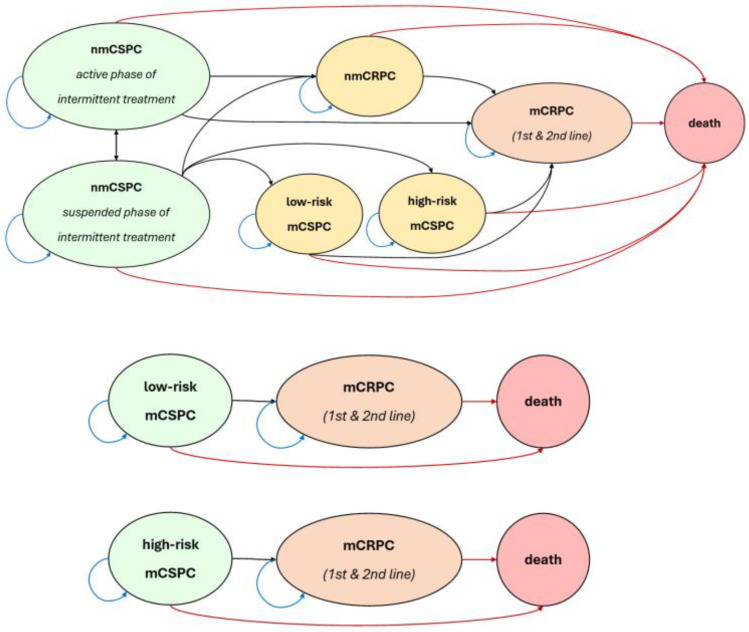
State transition diagram.

Following guidelines and recent PCa literature, we include the nmCSPC and mCSPC states. We stratify mCSPC into two separate states, low-risk/low-volume and high-risk/high-volume disease, taking advantage of stratified trial evidence while also following CUA guidelines, which recommend risk stratification due to a worse prognosis faced by high-risk/volume patients in the CHAARTED and LATITUDE trials [15,16]. For example, high-risk criteria include the presence of visceral metastases, ≥4 bone lesions, or Gleason score ≥8 [16]. In addition to the CSPC states, we have nmCRPC and mCRPC, including two lines of treatment in mCRPC. Note that there is no recovery stage in the model as there is currently no recognized evidence of complete remission of advanced PCa after treatment. We depict the health states we simulate in Figure 1.

Table 1 lists the randomized control trials we used as input to estimate model parameters [2,3,5,6,7,8,9,10,11,12,13,14,19,20,21,22,23,24,25]. All analyzed treatments are in combination with ADT. We also include enzalutamide use in nmCSPC due it its recent approval by Health Canada [26] and our expectation for its inclusion in upcoming clinical guidelines. These criteria leave us with 11 sequences for patients starting in nmCSPC, 20 treatment sequences for patients starting in low-risk mCSPC, and 26 sequences for patients starting in high-risk mCSPC. A list of all 57 sequences is presented in Section S1 of the Supplementary File S1.

Note that we do not include observational studies or other real-world evidence data as data inputs due to a lack of high-quality, large-sample analogs comparable to the randomized trials we included. This choice strengthens internal consistency and confidence in our results while also limiting the external generalizability of our results to patient groups and settings that differ substantially from randomized clinical trials, such as patients with serious comorbidities. We discuss the implications of this issue further in Section 4.

### 2.2. Model Structure

For each treatment and health state, the model inputs are progression-free survival (PFS) and overall survival (OS) Kaplan–Meier (KM) survival curves of relevant trials. After applying the Guyot et al. (2012) algorithm [27] for the KM survival curves of each treatment–state pair, we estimate three transition parameters: (1) probability of progression, (2) probability of death pre-progression, and (3) probability of death post-progression.

For each treatment sequence, we use a Monte Carlo Markov model, applying our estimated parameters to simulate the paths of 200,000 patients (i.e., microsimulation) over a 15-year horizon of 1-month periods. Note that since we lack reliable subgroup data, all simulated patients are assumed to be identical in terms of covariates like age or comorbidity. We model a discrete-time time-homogeneous semi-Markov process, where transition probabilities may depend on time spent in a health state but not on total time in advanced PCa. We use a 1.5% annual discount rate for both health benefits and financial costs, following CDA guidelines [28].

We programmed our model in MATLAB (R2023b). Also, WebPlotDigiter (v4.6) was used for digitizing Kaplan–Meier survival curves, and R (v4.3.2) was used for data cleaning and implementing the Guyot et al. (2012) algorithm [27].

### 2.3. Novel Parameter Estimation Algorithm

Our goal of modeling treatments in sequence introduces a statistical challenge that we tackle with a novel parameter estimation algorithm. This subsection features a limited technical overview of the problem and our solution.

To demonstrate the issue, suppose we wish to model a treatment sequence of treatment X in mCSPC followed by treatment Y in mCRPC. Suppose we use data from trial T_X_ that investigates treatment X within mCSPC with follow-up treatment Z in mCRPC and data from trial T_Y_ that investigates treatment Y within mCRPC. Since patients in T_X_ received Z and not Y in mCRPC, we need to separate T_X_ outcomes between events that occurred in mCSPC and those that occurred in mCRPC so that we can remove the latter. However, we cannot tell which events (namely deaths) in T_X_ happened pre- or post-progression to mCRPC, leading to a so-called “parameter identification problem.” This means that even in an ideal setting without statistical imprecision or noise, we can only “partially identify” the parameters of a set, with multiple options all being equally feasible.

Our solution is to specify a flexible, functional form on the transition parameters directly and then perform a grid search over a range of feasible values to find the parameters whose resulting (population-level) survival curves best fit the trial KM curves. Our algorithm provides a robust, flexible, and computationally efficient way to choose the model parameters which best fit the trial evidence.

The functional form of our transition probabilities involves four values (α β γ and δ), plus *t* representing the number of months spent in a health state. For each health state *s,* progressed state *p*, and death state *d,* we impose assumptions of constant pre- and post-progression probabilities while allowing for a flexible time trend of progression probability by allowing β to be positive or negative. The functional form of transition probabilities is as follows, with our algorithm choosing the values of (α β γ and δ) whose resulting survival curves provide the best fit to the real survival curves:

P(*s* → *p*) = α(1 + β)^(t−1)^

P(*s* → *d*) = γ

P(*p* → *d*) = δ

Our functional form strikes a balance between freedom and restrictiveness by using a few credible assumptions. With overly weak restrictions, the parameters would be free to take non-credible forms, such as abrupt shifts, and overfit the survival curves. With overly strong restrictions, there would be a risk of misspecifying the real transition probabilities and achieving a poor fit with the empirical survival curves.

Our method also advances analysis in the nascent field of estimating transition parameters for treatment sequences using survival curves. One existing work uses a piecewise exponential functional form but relies on visual inspection to select the best values, which is unreliable and cumbersome [29]. Instead, we optimize by minimizing mean-squared error, allowing for richer and more objective comparisons of feasible values. Another work overcomes the identification issue by assuming constant transition probabilities, which is simply too restrictive to be credible for our setting [30]. Instead, we achieve good fit to empirical survival curves by allowing time-varying progression. Finally, recent work has extended network meta-analysis to the issue of treatment sequences, overcoming partial identification by imposing assumptions on the form of transition probabilities to allow precise identification [31]. These identifying assumptions are not as simple as constant transition probabilities, which makes it more difficult to assess whether they are overly restrictive and how. Nevertheless, we are cautious about any method that assumes full knowledge of the transition probability specification. Therefore, we opt to use our method since it is the first to allow for the expression of greater uncertainty around transition probabilities in our setting while still allowing for a good fit with survival curves. However, a potential limitation of our method compared to existing ones is that we express too much uncertainty and allow transition parameters to vary in ways that we would ultimately deem non-credible. So far, we have not seen evidence of this issue in the setting of this paper.

In the survival curves analyzed for this article, the algorithm delivered comparable mean squared error to fitting using Weibull and Lognormal functions, as shown in Section S4.2 of Supplementary File S1. Also, we present our algorithm in more detail in Section S3 of Supplementary File S1, including methodological justification and further discussion of related works [29,30,31,32,33]. All transition probability parameters are available in Section S4.1 of the Supplementary File S1.

### 2.4. Utility Values

We use state-specific utility values to calculate the clinical benefit of treatments in QALYs. We report state-specific utility values in Table 2, where utility ranges from 0 at death to 1 at ideal health [34,35,36,37]. We interpolated nmCRPC utility from our mCSPC and mCRPC utility values using FACT-P quality-of-life questionnaire results [36]. We also apply constant treatment-specific disutilities to reflect adverse event risk from each treatment [19,38,39], mainly using calculations from Sathianathen et al. (2024) [38].

### 2.5. Treatment Costs

We examined direct drug costs, monitoring and administration costs, and end-of-life care costs. To best fit the Canadian public payer context, we incorporate direct drug costs using the RAMQ List of Medications from August 2024 [43] and estimates from Cancer Care Ontario [40,41]. We also used Cancer Care Ontario estimates of monthly IV and non-IV advanced PCa monitoring and administration costs [40]. Finally, we used Ontario estimates for PCa end-of-life care costs, totaling CAD 17,391 over the terminal 12 months of life for progressed mCRPC patients [42]. Also, we estimated monthly ADT cost by averaging the monthly costs of degarelix, leuporide, goserlein, and triptorelin, plus the cost of prednisone. We report monthly drug costs in Table 2 [40,41,42,43]. Note that ADT and ARPIs are taken each period, except for in nmCSPC, while the chemotherapeutic treatments of cabazitaxel and docetaxel continue for a fixed number of cycles, which we take from trial protocols [7,20,21,23,25,44].

In nmCSPC, costs reflect the fact that patients take ADT or enzalutamide+ADT intermittently as per the EMBARK trial protocol [2]. Each active phase of treatment lasts 9 months with full per-period drug costs in each month. Each suspended phase of treatment incurs only non-IV monitoring costs, and we model the length of time spent in suspended phases of treatment using a beta distribution fitted onto relevant EMBARK trial statistics [2]. We report the parameters for each treatment’s time-in-suspended-treatment probability distribution in Section S2 of Supplementary File S1. Additionally, we used the estimate from Francini et al. (2018) [45] of 65.1% of patients who transition from nmCSPC to mCSPC entering low-risk mCSPC, with the rest entering high-risk mCSPC.

### 2.6. Results Description

For each treatment sequence, all reported outcomes represent mean values averaged over the 200,000 simulated patient paths and discounted over the 15-year horizon period with an annual discount rate of 1.5%. We report incremental outcomes in terms of their difference over a “no ARPI” reference sequence.

We report our cost-effectiveness analysis results via mean net health benefit (NHB). NHB is mean benefit minus cost, with a cost-effectiveness threshold (CET) expressing the opportunity cost of treatment.

For clarity, we present the calculation of incremental NHB below:

$$NHB=Benefit-\frac{Cost}{CET}$$

Units

NHB: QALYs (or LYs)

Benefit: Incremental QALYs (or LYs)

Cost: Incremental CAD 

CET: $\frac{CAD}{QALYs\left(or\;LYs\right)}$

We opt to report incremental NHB instead of an incremental cost-effectiveness ratio (ICER), in line with contemporary recommendations on the use of net health benefit or net monetary benefit instead of an ICER in reporting results [46]. This is especially salient in our case, where the large number of interventions under comparison leads NHB to have a clearer interpretation than ICER.

### 2.7. Sequence Description

First, within each health state, we define “ARPI use” as prescription of an ARPI, either exclusively or alongside another non-ARPI treatment such as docetaxel chemotherapy. Following NCCN and CUA guidelines [15,17] that reference a risk of ARPI cross-resistance, we exclude any treatment sequences with ARPI rechallenge. We define a sequence as “early ARPI use” if it assigns ARPI use in the starting state and “late ARPI use” if it assigns ARPI use in a later state instead. To keep our analysis and exposition clear, we only examine nmCSPC-starting “late ARPI use” sequences where patients in mCSPC and nmCRPC receive the same ARPI treatment.

## 3. Results

**Figure 2:**
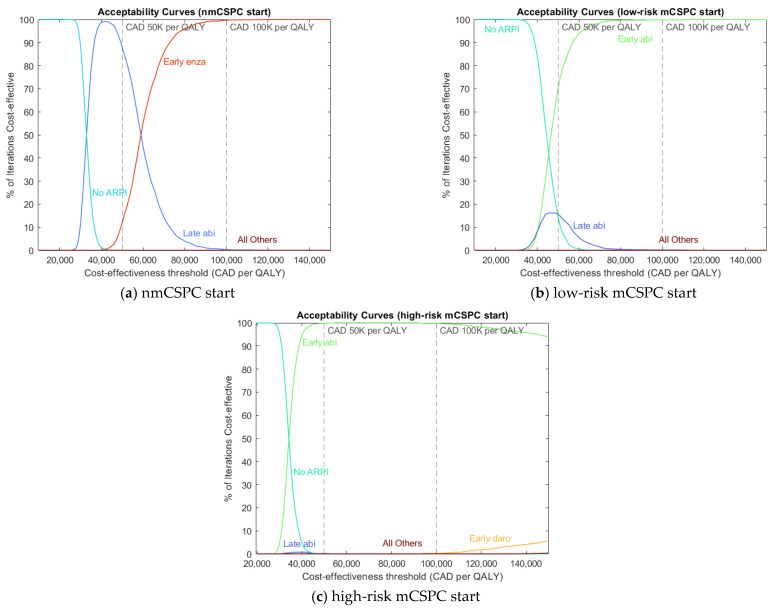
Acceptability curves for (**a**) patients starting in nmCSPC, (**b**) patients starting in low-risk mCSPC, and (**c**) patients starting in high-risk mCSPC.

We display the most efficacious and cost-effective treatment sequences in Table 3 and Table 4, respectively. We also display acceptability curve (AC) graphs with sequences grouped by ARPI and timing of APRI use for readability in Figure 2. These AC graphs have the dual purpose of showing how our cost-effectiveness results vary over different CETs and showing the variance of these results at each CET. To strike a balance that achieves each aim, we sample 2000 iterations of 100 patients each and use the mean benefit and cost achieved in each iteration to calculate AC values. Note that there is no need to calculate ICER as part of generating AC values. AC graphs showing individual treatment sequences and pairwise treatment comparisons, as well as benefit–cost plots, are available in Section S5 of Supplementary File S1. The model output used to generate the main results are available in Supplementary Files S2, S3, and S4.

We find that ARPIs have more efficacy than non-ARPI treatments and that ARPIs have more efficacy the earlier they are taken, both in terms of LYs and QALYs. We find that for Canadian funding levels of CAD 100K per QALY, early ARPI use is the most cost-effective choice. For a CET of CAD 50K per QALY, early ARPI use is the most cost-effective choice for patients starting in mCSPC, while later ARPI use is most cost-effective for patients starting in nmCSPC. Note that for CET of CAD 50K per QALY, the advantage of early ARPI use is small for low-risk mCSPC patients.

The results also show that as the payer’s funding availability rises (falls), early ARPI use becomes relatively more (less) cost-effective, as evident in the AC graphs. The underlying reason for this pattern is that clinical benefit and financial cost are both high for early ARPI use, both medium for late ARPI use, and both low for no ARPI use. At higher CETs, around CAD 100K per QALY, there is enough funding to prefer the “high benefit and high cost” option of early ARPI use, but as CETs fall, the opportunity cost of early ARPI use eventually outweighs the benefit. The AC graphs show that for mCSPC-starting patients, no ARPI use becomes more cost-effective for low CETs (below CAD 40K per QALY), although late ARPI use shows limited cost-effectiveness in low-risk mCSPC at CETs around CAD 50K per QALY. For nmCSPC-starting patients, the “medium” option of late ARPI use is most cost-effective for CETs around CAD 50K per QALY, with no ARPI use winning out at lower CETs.

In terms of specific treatment sequences, we find that the most efficacious treatments involve the early use of enzalutamide or apalutamide. By contrast, we find that the most cost-effective treatment sequences in mCSPC-starting patients involve early abiraterone use. For nmCSPC-starting patients, later abiraterone use or early intermittent enzalutamide use is cost-effective, depending on the CET. Note that the only ARPI currently evaluated in nmCSPC is enzalutamide, so the comparison between enzalutamide and other ARPIs in this health state will have to follow future trial evidence. We find that abiraterone excels in cost-effectiveness because it provides roughly comparable state-specific health outcomes to the other ARPIs while having a direct drug cost that is over three-and-a-half times smaller. Since this result is largely driven by drug costs, it may not apply to settings with relative or absolute drug prices that are substantially different from the contemporary Canadian context.

### Sensitivity Analysis

We perform sensitivity analyses along three dimensions: time-horizon length, drug price, and payer perspective. First, we examine the cases of 10-year and 20-year time horizons. Second, we examine the case where the direct drug costs of apalutamide, darolutamide, and enzalutamide are lowered to those of abiraterone. Third, we examine the perspectives of a US public payer (namely Veterans Affairs), a US private payer (paying wholesale drug prices), and a UK public payer.

Our results are sensitive to choices of alternate time horizons, especially shorter ones. Increasing the time horizon to 20 years strengthens the advantage of early ARPI use while shortening the time horizon to 10 years leads to late and no ARPI use to become more cost-effective. The underlying reason for this pattern is that early ARPI use incurs financial costs upfront, while the differential increase in longevity is truncated (extended) for shorter (longer) time horizons. The implications are that early ARPI use is likely more cost-effective for patient groups with longer expected longevity, as well as payers who are more forward-looking. Detailed results are presented in Section S5.4 of the Supplementary File S1.

We find that equalizing ARPI prices to the Canadian price of abiraterone leads to the early use of apalutamide, darolutamide, and enzalutamide to become most cost-effective for different starting states. This is in stark contrast to the base case where abiraterone is most cost-effective for most CETs and starting states. Instead, enzalutamide is now best for nmCSPC and low-risk mCSPC, darolutamide is best for high-risk mCSPC with a CET of CAD 50K per QALY, and apalutamide is best for high-risk mCSPC with a CET of CAD 100K per QALY. This sensitivity analysis confirms that abiraterone is most cost-effective in the base results primarily due to its substantially lower cost and not due to a superior clinical benefit. The main implication is that as other ARPI prices likely fall in the future due to off-patent status, early ARPI use will likely become more cost-effective, and there will be a wider variety of cost-effective drugs across starting states. Detailed results are presented in Section S5.5 of the Supplementary File S1.

To model a US public and private payer perspective, we use US Veterans Affairs drug prices and average wholesale drug prices, respectively, as calculated by Yoo et al. (2023) [47]. In addition, we adjust the annual discount rate to 3% to meet US standards and use the Bank of Canada’s annual 2024 exchange rate of 1.3698 CAD per USD to compare CETs [48]. To model a UK public payer perspective, we use UK public drug prices, as calculated by Sathianathen et al. (2024) [38]. In addition, we adjust the annual discount rate to 3.5% to meet UK standards and use the Bank of Canada’s annual 2024 exchange rate of 1.7504 CAD per GBP to compare CETs [48]. We report the US and UK prices used in Section S5.6 of the Supplementary File S1.

**Figure 3:**
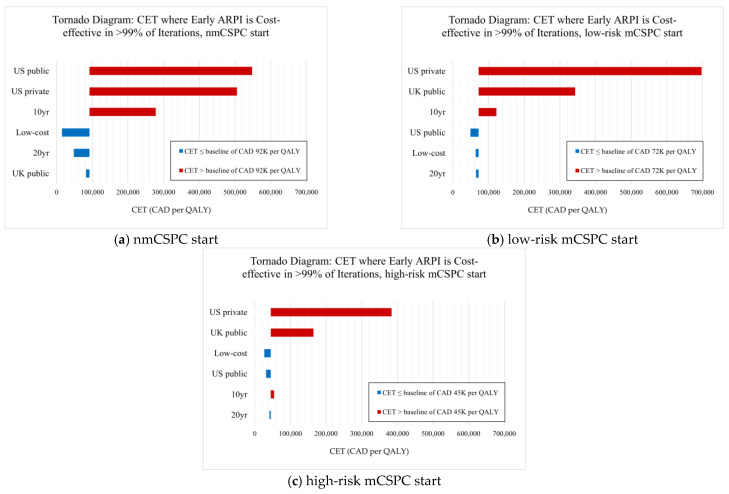
Tornado diagram examining sensitivity scenarios for (**a**) patients starting in nmCSPC, (**b**) patients starting in low-risk mCSPC, and (**c**) patients starting in high-risk mCSPC.

Our results are sensitive to alternate geographic and payer perspectives, mostly due to differences in prices faced. As Figure 3 shows, US and UK payer perspectives lead to early ARPI use being substantially less cost-effective, requiring prohibitively high CETs of up to CAD 300K per QALY or higher to justify early ARPI use for some starting health states. With the US public payer perspective, we find that abiraterone use (late in nmCSPC, early in low-risk/high-risk mCSPC) is the most cost-effective option since abiraterone prices are relatively lower compared to Canada, but other ARPIs are relatively more expensive. From the US private payer perspective, we find that no APRI use is the most cost-effective option for all starting states due to substantially higher prices for all drugs, especially ARPIs. For the UK public payer perspective, we find that no ARPI use is most cost-effective for mCSPC starting patients due to abiraterone being relatively more expensive than in Canada, while no ARPI use is cost-effective for nmCSPC patients due to a lower CET than in Canada being enough to disfavor early ARPI use.

Focusing on CETs in particular, our results are sensitive to choices of different CETs found in non-Canadian settings. A standard US payer CET is approximately USD 100K per QALY, equivalent to CAD 137K per QALY, which is larger than the high-end Canadian CET [47]. Our base and US sensitivity results are robust to alternative choices of Canadian or US CETs since early ARPI use becomes cost-effective at lower thresholds for Canadian payers and mCSPC-starting US public payers or much higher thresholds for US private payers and nmCSPC-starting US public payers. Moreover, a standard UK public payer CET of GBP 20–30K per QALY is equivalent to only CAD 35–50K per QALY, just overlapping with the lower end of the Canadian CET range. Therefore, if we were to apply a UK CET to a Canadian payer, then late ARPI use or no ARPI use would be most cost-effective. Similarly, applying a Canadian CET to the UK setting changes the nmCSPC-starting results, where early enzalutamide use becomes cost-effective for nmCSPC patients due to enzalutamide’s similar price in the UK.

We present tornado diagrams for each starting state and sensitivity scenario below in Figure 3. First, we find the lowest CET at which early ARPI use is cost-effective for >99% of iterations in our base results, representing a CET baseline for supporting early ARPI use. There is a different base CET for each starting health state at which early ARPI use becomes cost-effective, such as CAD 92K per QALY for nmCSPC-starting patients. We then find the analogous CET for each sensitivity scenario, where a higher (red) CET means that early ARPI use is only cost-effective at higher funding levels, while a lower (blue) CET means that early ARPI use is cost-effective at even lower funding levels than in the base case.

## 4. Discussion

The introduction of ARPIs has broadened the set of options for advanced PCa management. Recent research examining the cost-effectiveness of ARPIs using contemporary trial evidence includes Yoo et al. (2023) [47], Sathianathen et al. (2024) [38], and Saad et al. (2022) [49], with the first two papers primarily examining the US public payer context and the last paper examining the Canadian public payer context. Each of these articles compares different interventions at the mCSPC stage without stratifying for risk, as in our paper. To the best of our knowledge, our paper is also the first to compare treatment sequences for advanced PCa care using a Markov model and the first to incorporate the nmCSPC health state.

First, we find that using ARPIs in nmCSPC and mCSPC when patients first enter advanced PCa is the most efficacious option, generating high clinical benefit. We find that for payers facing Canadian price levels, early ARPI use is also the most cost-effective option. Also, we conclude early ARPI use is relatively more (less) cost-effective for payers with larger (lower) funding levels or longer (shorter) time horizons.

We find that abiraterone for mCSPC and intermittent enzalutamide for nmCSPC are the most cost-effective ARPIs for advanced PCa management in the Canadian public payer context. We also find that abiraterone is currently the most cost-effective ARPI in the contemporary Canadian context due to its similar clinical benefit and substantially lower cost compared to other ARPIs. We conclude that if other ARPIs were to have costs comparable to abiraterone, each of them would be most cost-effective for advanced PCa care. This result allows us to predict that as patents expire and prices likely fall, early use of apalutamide, darolutamide, and enzalutamide may become the most cost-effective options for mCSPC-starting patients.

Our findings largely align with and extend the findings of Saad et al. (2022) [49] and Yoo et al. (2023) [47]. Saad et al. (2022) [49] do not examine abiraterone, but our papers share the result that enzalutamide is the most efficacious treatment and among the most expensive treatments for (low-risk) mCSPC patients. Yoo et al. (2023) [47] find that abiraterone use in mCSPC is most cost-effective, aligning with our results. This is because their US public payer perspective shares the same generic abiraterone pricing as our base setting. By contrast, Sathianathen et al. (2024) [38] find that no ARPI use is most cost-effective in mCSPC for a US public payer. This is because they use far higher brand pricing for abiraterone, aligning closer to the US private payer case in our sensitivity analysis.

Despite the differences in absolute drug prices between American and Canadian public payer perspectives, our findings for mCSPC-starting patients align with the findings of Yoo et al. (2023) [47], as well as the sensitivity analysis results of Sathianathen et al. (2024) [38] that examine U.S. generic abiraterone prices.

Our sensitivity analysis examining US and UK perspectives finds that drug prices and local CETs in alternate settings are likely to affect our results, albeit in a predictable way. For example, US private payers face higher ARPI prices, so no-ARPI sequences are the most cost-effective. By contrast, UK public payers face higher prices for abiraterone and lower prices for enzalutamide, so as the underlying patterns in our model would predict, early ARPI use is more cost-effective for nmCSPC patients and less cost-effective for mCSPC patients. Since the reverse is true for US public payers in terms of relative abiraterone and enzalutamide prices, early ARPI use is relatively less cost-effective for nmCSPC patients and more cost-effective for mCSPC patients. Finally, as CETs (adjusted for exchange rates) grow or fall by jurisdiction, early ARPI use becomes more or less cost-effective, respectively.

The implications of our findings for clinical practice are that ARPIs should be used early in advanced PCa, barring contraindications against ARPIs. For payers, the implication of our findings is that in a Canadian public payer setting, ARPIs should be funded for use by advanced PCa patients without prior ARPI use, especially in CSPC. These findings should be interpreted with caution for payers facing relatively higher prices for abiraterone or enzalutamide, such as US private payers or UK public payers.

Our model has several constraints and limitations that may limit its generalizability. Since we rely on randomized clinical trials to model clinical outcomes, our results should be interpreted with caution for patient groups that differ significantly from trial samples, such as patients with comorbidities which do not typically meet trial eligibility criteria. However, we do not have enough information to confidently assess the direction in which our results may be biased by using randomized trials instead of observational study data, so we refrain from attempting to characterize our results as either upper or lower bounds with respect to real patient population outcomes. In addition, our data sources do not provide survival curves for subgroups of patients stratified by age or health status (beyond low/high-risk mCSPC status), preventing us from providing a more granular analysis of patient subpopulations. We hope to conduct such work if more granular data becomes available.

Also, since our base case considers public payer drug costs, clinical guidelines, and oncological cost-effectiveness thresholds that are relevant to Canada, our conclusions may not hold for other settings and regions with substantively different drug costs, guidelines, or regulatory features such as private payers or pharmacy benefit managers. Another limitation of our model is that our conclusions will require updating to reflect future changes in available drugs, indications, drug prices, and expiring drug patents. Moreover, we only consider the public and private payer perspectives, not alternate options such as societal perspectives that incorporate wider effects of illness on caregivers, workplaces, and other societal stakeholders.

In addition, we use CUA and NCCN clinical guidelines to select our treatment sequences, which limits our results to settings that have clinical practice and payer reimbursement criteria aligned with CUA and NCCN guidelines for advanced PCa treatment, particularly with respect to ARPI use. Another limitation is that we do not model alternate real-world treatment sequences, such as those with ARPI rechallenge, due to a lack of relevant trial evidence which we can use to estimate model parameters. By not modeling these sequences, it is possible that we are missing efficacious or cost-effective sequences which would form the headline results. There is a good opportunity for future research to fill this gap, especially as high-quality, real-world evidence of ARPI use becomes available.

Finally, our study did not include other less-used treatments in the later stages of the mCRPC state, such as PARP inhibitors (for those carrying HDRR mutations), radium, or lutetium. We also did not account for the possible different monitoring and management options of prednisone-related effects when patients receive long-term abiraterone.

## 5. Conclusions

Our goal of examining treatment sequences introduced a methodological challenge in the form of an identification problem. In response, we developed a novel parameter estimation algorithm that enabled us to estimate transition probabilities that align with trial data. There is an opportunity for future theoretical work on how to tackle the identification problem for Markov models.

Finally, the rapidly changing trial and regulatory landscape will provide future opportunities to refresh this paper’s analysis. The expected completion of the FINITE trial in 2025 [50,51] will provide results on abiraterone use in nmCSPC. In addition, the expected expiry of the Canadian enzalutamide patent in 2026 [26] will likely lead to changes in the ARPI drug cost landscape [52,53].

This article is a revised and expanded version of a paper entitled “Cost-Effectiveness Analysis of Contemporary Advanced Prostate Cancer Management: A Markov Model for the Canadian Context”, which was presented by ISPOR Europe 2024 in Barcelona, Spain, in November 2024 [54].

## Figures and Tables

**Table 1:** List of trials, treatments, and health states used.

Trial	Treatments	Health States	Sources
AFFIRM	Enzalutamide post docetaxel	mCRPC	[11]
ARAMIS	Darolutamide	nmCRPC	[12]
	ADT alone	nmCRPC	
ARANOTE	Darolutamide	low-risk mCSPC	[6]
		high-risk mCSPC	
ARASENS	Darolutamide and docetaxel	low-risk mCSPC	[7]
		high-risk mCSPC	
COU-AA-301	Abiraterone post docetaxel	mCRPC	[9]
COU-AA-302	Abiraterone	mCRPC	[10]
EMBARK	Enzalutamide (intermittent)	nmCSPC	[2]
	ADT alone (intermittent)	nmCSPC	
ENZAMET	Enzalutamide	low-risk mCSPC	[8]
		high-risk mCSPC	
	Enzalutamide and docetaxel	low-risk mCSPC	
		high-risk mCSPC	
	Docetaxel	high-risk mCSPC	
	ADT alone	low-risk mCSPC	
		high-risk mCSPC	
FIRSTANA	Docetaxel	mCRPC	[19]
GETUG-AFU 15	Docetaxel	high-risk mCSPC	[20]
	ADT alone	high-risk mCSPC	
PEACE-1	Abiraterone and docetaxel	low-risk mCSPC	[4]
		high-risk mCSPC	
	Docetaxel	high-risk mCSPC	
PRESIDE	Docetaxel	mCRPC	[21]
PREVAIL	Enzalutamide	mCRPC	[22]
PROSELICA	Cabazitaxel post docetaxel	mCRPC	[23]
PROSPER	Enzalutamide	nmCRPC	[13]
	ADT alone	nmCRPC	
SPARTAN	Apalutamide	nmCRPC	[14]
	ADT alone	nmCRPC	
STAMPEDE	Abiraterone	low-risk mCSPC	[5]
		high-risk mCSPC	
		nmCRPC	[24]
	ADT alone	low-risk mCSPC	[5]
		high-risk mCSPC	
TITAN	Apalutamide	low-risk mCSPC	[3]
		high-risk mCSPC	
TROPIC	Cabazitaxel post docetaxel	mCRPC	[25]

**Table 2:** Utility and cost parameters.

Health State	State-Specific Utility (Annual)	Sources
nmCSPC	0.95	[34]
mCSPC (low- and high-risk)	0.85	[35]
nmCRPC	0.9	[35,36,37]
mCRPC	0.75	[37]
Progressed mCRPC	0.6	[37]
**Treatment**	**Treatment-Specific Disutility (Annual)**	**Sources**
Abiraterone	−0.021	[38]
Apalutamide	−0.019	[38]
Cabazitaxel	−0.042	[19,38,39]
Darolutamide	−0.019	[38]
Docetaxel	−0.042	[38]
Enzalutamide	−0.022	[38]
**Treatment**	**Cost (CAD, Monthly)**	**Sources**
ADT	322	[40,41]
Abiraterone	919	[41]
Apalutamide	3401	[41]
Cabazitaxel	4134	[42]
Darolutamide	3401	[41]
Docetaxel	103	[40]
Enzalutamide	3401	[41]
Non-IV management	92	[40]
IV management	455	[40]
End-of-life	1449	[43]

**Table 3:** Most efficacious treatment sequences.

Measure	Starting Health State	Rank	Incremental Benefit	Total Benefit	Treatment Sequence (nmCSPC, nmCRPC/mCSPC, mCRPC) * or (mCSPC, mCRPC) ^†^	ARPI Use
QALYs	nmCSPC	1	3.29 QALYs	10.87 QALYs	(enza, adt, doce) *	early
		2	3.18 QALYs	10.75 QALYs	(enza, adt, doce then caba) *	early
		3	1.99 QALYs	9.57 QALYs	(adt, abi, doce) *	late
	low-risk mCSPC	1	2.23 QALYs	7.34 QALYs	(enza, doce) ^†^	early
		2	2.02 QALYs	7.13 QALYs	(abi, doce) ^†^	early
		3	1.93 QALYs	7.03 QALYs	(enza, doce then caba) ^†^	early
	high-risk mCSPC	1	3.02 QALYs	6.44 QALYs	(apa, doce) ^†^	early
		2	2.71 QALYs	6.13 QALYs	(apa, doce then caba) ^†^	early
		3	2.22 QALYs	5.64 QALYs	(doce + daro, doce) ^†^	early
LYs	nmCSPC	1	3.03 LYs	11.82 LYs	(enza, adt, doce) *	early
		2	2.82 LYs	11.62 LYs	(enza, adt, doce then caba) *	early
		2	2.06 LYs	10.86 LYs	(adt, abi, doce) *	late
	low-risk mCSPC	1	2.65 LYs	9.30 LYs	(enza, doce) ^†^	early
		2	2.37 LYs	9.02 LYs	(abi, doce) ^†^	early
		3	2.11 LYs	8.77 LYs	(enza, doce then caba) ^†^	early
	high-risk mCSPC	1	3.57 LYs	8.18 LYs	(apa, doce) ^†^	early
		2	3.02 LYs	7.63 LYs	(apa, doce then caba) ^†^	early
		3	2.74 LYs	7.35 LYs	(doce + daro, doce) ^†^	early

**Note:** * indicates nmCSPC-starting sequence. ^†^ indicates mCSPC-starting sequence.

**Table 4:** Most cost-effective treatment sequences.

Starting Health State	Cost Effectiveness Threshold	Rank	Incremental NHB (QALY)	Total NHB (QALY)	Total Benefit (QALY)	Total Cost (CAD)	Treatment Sequence (nmCSPC, nmCRPC/mCSPC, mCRPC) * or (mCSPC, mCRPC) ^†^	ARPI Use
nmCSPC	CAD 50K per QALY	1	0.68	7.65	9.57	95,780	(adt, abi, doce) *	late
		2	0.45	7.41	10.87	172,579	(enza, adt, doce) *	early
		3	0.26	7.22	9.41	109,546	(adt, abi, doce then caba) *	late
	CAD 100K per QALY	1	1.87	9.14	10.87	172,579	(enza, adt, doce) *	early
		2	1.66	8.93	10.75	181,883	(enza, adt, doce then caba) *	early
		3	1.34	8.61	9.57	95,780	(adt, abi, doce) *	late
low-risk mCSPC	CAD 50K per QALY	1	0.15	4.59	7.13	126,870	(abi, doce) ^†^	early
		2	0	4.44	5.11	33,505	(adt, doce) ^†^	none
		3	0.00	4.44	5.58	57,168	(adt, abi) ^†^	late
	CAD 100K per QALY	1	1.09	5.86	7.13	126,870	(abi, doce) ^†^	early
		2	0.58	5.35	6.84	148,756	(abi, doce then caba) ^†^	early
		3	0.24	5.01	5.58	57,168	(adt, abi) ^†^	late
high-risk mCSPC	CAD 50K per QALY	1	0.65	3.60	5.45	92,454	(abi, doce) ^†^	early
		2	0.06	3.01	4.52	75,713	(doce + abi, doce) ^†^	early
		3	0.00	2.96	3.85	44,807	(adt, abi) ^†^	late
	CAD 100K per QALY	1	1.34	4.52	5.45	92,454	(abi, doce) ^†^	early
		2	0.89	4.08	5.64	156,020	(doce + daro doce) ^†^	early
		3	0.71	3.89	5.06	117,143	(abi, doce then caba) ^†^	early
**Starting Health State**	**Cost Effectiveness Threshold**	**Rank**	**Incremental NHB (LY)**	**Total NHB (LY)**	**Total Benefit (LY)**	**Total Cost (CAD)**	**Treatment Sequence** (nmCSPC, nmCRPC/mCSPC, mCRPC) * **or (mCSPC, mCRPC) ^†^**	**ARPI Use**
nmCSPC	CAD 50K per LY	1	0.76	8.95	10.86	95,780	(adt, abi, doce) *	late
		2	0.21	8.40	10.59	109,546	(adt, abi, doce then caba) *	late
		3	0.18	8.37	11.82	172,579	(enza, adt, doce) *	early
	CAD 100K per LY	1	1.60	10.10	11.82	172,579	(enza, adt, doce) *	early
		2	1.41	9.90	10.86	95,780	(adt, abi, doce) *	late
		3	1.31	9.80	11.62	181,883	(enza, adt, doce then caba) *	early
low-risk mCSPC	CAD 50K per LY	1	0.50	6.48	9.02	126,870	(abi, doce) **^†^**	early
		2	0.04	6.02	7.16	57,168	(adt, abi) **^†^**	late
		3	0	5.98	6.65	33,505	(adt, doce) **^†^**	none
	CAD 100K per LY	1	1.43	7.75	9.02	126,870	(abi, doce) **^†^**	early
		2	0.70	7.02	8.51	148,756	(abi, doce then caba) **^†^**	early
		3	0.27	6.59	7.16	57,168	(adt, abi) **^†^**	late
high-risk mCSPC	CAD 50K per LY	1	1.10	5.24	7.09	92,454	(abi, doce) **^†^**	early
		2	0.39	4.54	6.05	75,713	(doce + abi, doce) **^†^**	early
		3	0.08	4.23	7.35	156,020	(doce + daro, doce) **^†^**	early
	CAD 100K per LY	1	1.79	6.16	7.09	92,454	(abi, doce) **^†^**	early
		2	1.41	5.79	7.35	156,020	(doce + daro, doce) **^†^**	early
		3	0.92	5.29	6.05	75,713	(doce + abi, doce) **^†^**	early

**Note:** * indicates nmCSPC-starting sequence. ^†^ indicates mCSPC-starting sequence.

## Data Availability

The original contributions presented in this study are included in the article/Supplementary Materials. Further inquiries can be directed to the corresponding author.

## Supplementary Materials

The following supporting information can be downloaded at: https://www.mdpi.com/article/10.3390/curroncol32040240/s1, Supplementary File S1, including Section S1: List of Treatment Sequences; Section S2: Time-in-suspended-treatment Parameters; Section S3: Parameter Estimation Algorithm; Section S4: Transition Probability Parameter Estimates; Section S5: Additional Figures and Tables; Supplementary CSV files S2: nmcspc_results, S3: low_risk_mcspc_results, and S4: high_risk_mcspc_results with main numerical results for patients starting in nmCSPC, low-risk mCSPC, and high-risk mCSPC, respectively. References [2,27,29,30,31,32,33,45] are cited in the Supplementary Materials.

## Abbreviations

The following abbreviations are used in this manuscript:

PCa	Prostate cancer
ARPI	Androgen receptor pathway inhibitor
ADT	Androgen deprivation therapy
nmCSPC	Nonmetastatic castration-sensitive prostate cancer
mCSPC	Metastatic castration-sensitive prostate cancer
nmCRPC	Nonmetastatic castration-resistant prostate cancer
mCRPC	Metastatic castration-resistant prostate cancer
NCCN	National Comprehensive Cancer Network
CUA	Canadian Urological Association
CDA	Canada’s Drug Agency
PFS	Progression-free survival
OS	Overall survival
KM	Kaplan–Meier
NHB	Net health benefit
CET	Cost-effectiveness threshold
ICER	Incremental cost-effectiveness ratio
AC	Acceptability curve
abi	Abiraterone
apa	Apalutamide
caba	Cabazitaxel
daro	Daroluatmide
doce	Docetaxel
enza	Enzalutamide
