# Supplementary material for: Cost-Effectiveness Analysis of Contemporary Advanced Prostate Cancer Treatment Sequences
Source: Curr Oncol. 2025 Apr 20;32(4):240. doi: 10.3390/curroncol32040240 (PMC12025438; doi:10.3390/curroncol32040240)
Supplement: Supplementary file 1 [file curroncol-32-00240-s001.zip › curroncol-3529611-supplementary.pdf]

Supplementary Materials for *Cost-Effectiveness Analysis of Contemporary Advanced Prostate Cancer Treatment Sequences*

Overview

These supplementary materials have five sections: S1) List of Treatment Sequences, S2) Time-in-suspended Treatment Parameters, S3) Parameter Estimation Algorithm, S4) Transition Probability Parameter Estimates, and S5) Additional Figures and Tables.

S1 List of Treatment Sequences

Table S1: List of sequences for low-risk mCSPC-starting patients.

Abbreviated Sequence	mCSPC treatment	mCRPC treatment
(adt, abi)	ADT	Abiraterone
(adt, enza)	ADT	Enzalutamide
(adt, doce)*	ADT	Docetaxel
(adt, doce then abi)	ADT	Docetaxel then abiraterone
(adt, doce then caba)	ADT	Docetaxel then cabazitaxel
(adt, doce then enza)	ADT	Docetaxel then enzalutamide
(abi, doce)	Abiraterone	Docetaxel
(abi, doce then caba)	Abiraterone	Docetaxel then cabazitaxel
(apa, doce)	Apalutamide	Docetaxel
(apa, doce then caba)	Apalutamide	Docetaxel then cabazitaxel
(doce+abi, doce)	Docetaxel & abiraterone	Docetaxel
(doce+abi, doce then caba)	Docetaxel & abiraterone	Docetaxel then cabazitaxel
(daro, doce)	Darolutamide	Docetaxel
(daro, doce then caba)	Darolutamide	Docetaxel then cabazitaxel
(doce+daro, doce)	Docetaxel & darolutamide	Docetaxel
(doce+daro, doce then caba)	Docetaxel & darolutamide	Docetaxel then cabazitaxel
(doce+enza, doce)	Docetaxel & enzalutamide	Docetaxel
(doce+enza, doce then caba)	Docetaxel & enzalutamide	Docetaxel then cabazitaxel
(enza, doce)	Enzalutamide	Docetaxel
(enza, doce then caba)	Enzalutamide	Docetaxel then cabazitaxel

"No ARPI reference sequence" is marked with * symbol.

Table S2: List of sequences for high-risk mCSPC-starting patients.

Abbreviated Sequence	mCSPC treatment	mCRPC treatment
(adt, abi)	ADT	Abiraterone
(adt, enza)	ADT	Enzalutamide
(adt, doce)*	ADT	Docetaxel
(adt, doce then abi)	ADT	Docetaxel then abiraterone
(adt, doce then caba)	ADT	Docetaxel then cabazitaxel
(adt, doce then enza)	ADT	Docetaxel then enzalutamide
(abi, doce)	Abiraterone	Docetaxel
(abi, doce then caba)	Abiraterone	Docetaxel then cabazitaxel
(apa, doce)	Apalutamide	Docetaxel
(apa, doce then caba)	Apalutamide	Docetaxel then cabazitaxel
(doce, abi)	Docetaxel	Abiraterone
(doce, enza)	Docetaxel	Enzalutamide
(doce, doce)	Docetaxel	Docetaxel
(doce, doce then abi)	Docetaxel	Docetaxel then abiraterone
(doce, doce then caba)	Docetaxel	Docetaxel then cabazitaxel
(doce, doce then enza)	Docetaxel	Docetaxel then enzalutamide
(doce+abi, doce)	Docetaxel & abiraterone	Docetaxel
(doce+abi, doce then caba)	Docetaxel & abiraterone	Docetaxel then cabazitaxel
(daro, doce)	Darolutamide	Docetaxel
(daro, doce then caba)	Darolutamide	Docetaxel then cabazitaxel
(doce+daro, doce)	Docetaxel & darolutamide	Docetaxel
(doce+daro, doce then caba)	Docetaxel & darolutamide	Docetaxel then cabazitaxel
(doce+enza, doce)	Docetaxel & enzalutamide	Docetaxel
(doce+enza, doce then caba)	Docetaxel & enzalutamide	Docetaxel then cabazitaxel
(enza, doce)	Enzalutamide	Docetaxel
(enza, doce then caba)	Enzalutamide	Docetaxel then cabazitaxel

"No ARPI reference sequence" is marked with * symbol.

Table S3: List of sequences for nmCSPC-starting patients.

Abbreviated Sequence	nmCSPC treatment	nmCRPC treatment	low-risk mCSPC treatment	high-risk mCSPC treatment	mCRPC treatment
(adt, adt, doce)*	Intermittent ADT	ADT	ADT	ADT	Docetaxel
(adt, abi, doce)	Intermittent ADT	Abiraterone	Abiraterone	Abiraterone	Docetaxel
(adt, abi, doce then caba)	Intermittent ADT	Abiraterone	Abiraterone	Abiraterone	Docetaxel then cabazitaxel
(adt, apa, doce)	Intermittent ADT	Apalutamide	Apalutamide	Apalutamide	Docetaxel
(adt, apa, doce then caba)	Intermittent ADT	Apalutamide	Apalutamide	Apalutamide	Docetaxel then cabazitaxel
(adt, daro, doce)	Intermittent ADT	Darolutamide	Darolutamide	Darolutamide	Docetaxel
(adt, daro, doce then caba)	Intermittent ADT	Darolutamide	Darolutamide	Darolutamide	Docetaxel then cabazitaxel
(adt, enza, doce)	Intermittent ADT	Enzalutamide	Enzalutamide	Enzalutamide	Docetaxel
(adt, enza, doce then caba)	Intermittent ADT	Enzalutamide	Enzalutamide	Enzalutamide	Docetaxel then cabazitaxel
(enza, adt, doce)	Intermittent enzalutamide	ADT	ADT	ADT	Docetaxel
(enza, adt, doce then caba)	Intermittent enzalutamide	ADT	ADT	ADT	Docetaxel then cabazitaxel

"No ARPI reference sequence" is marked with * symbol.

S2 Time-in-suspended-treatment Parameters

Following the protocol of the EMBARK trial[2], we model intermittent ADT and enzalutamide treatment by having nmCSPC patients start with an active phase lasting nine months. However, some trial patients required multiple rounds of active treatment, requiring us to model the probability distributions of exiting the suspended phase and re-entering the active phase of treatment. The EMBARK trial[2] provides some summary statistics on the number of months patients spent in the suspended phase of intermittent treatment. We provide them in the following table:

Table S4: Time-in-suspended-treatment inputs.

Treatment	Min (mo)	Max (mo)	Median (mo)	Percent suspended at 24mo
ADT	3.4	83.0	16.8	32.1%
Enzalutamide	5.7	87.9	20.2	43.9%

Abbreviations: mo: months

For each treatment, we fit a beta distribution to the median months and “percent suspended at 24mo” values, scaling and shifting the domain from $[0,1]$to $[min, max]$ the found in the trial for each treatment. Call the estimated Beta parameters α and β for the first and second shape parameters, respectively. Define the inputted min and max months for each treatment t as l_t and u_t, respectively. Then the probability for each patient of exiting the suspended phase of treatment t by month m (conditional on remaining in nmCSPC) is given by

$$F_t(m) = l_t + (u_t - l_t)F_{\text{Beta}}(\alpha, \beta)$$

We estimate the following Beta parameters for our model:

Table S5: Time-in-suspended-treatment parameter estimates.

Treatment	l_t	u_t	α	β
ADT	3.4	83.0	2.00	7.97
Enzalutamide	5.7	87.9	2.00	7.53

S3 Parameter Estimation Algorithm

S3.1 Identification Problem

Let us begin with a theoretical discussion of the parameter identification problem when estimating transition probabilities for sequences of counterfactual treatments. Suppose there are three health states $\{s, p, d\}$ arranged in order of increasing disease progression and ending with death. For example, these states could represent {mCSPC, mCRPC, death}. Suppose that the only transitions that can occur are $s \to p$, $s \to d$, and $p \to d$. Suppose we have Kaplan-Meier (KM) survival curves from a trial T_X where patients receive treatment X in s and treatment Z in p. Suppose we also have a KM curve from another trial T_Y where patients receive treatment Y in p.

Our goal is to model the counterfactual sequence of patient outcomes from X in s followed by Y in p. In turn, we need to combine the s evidence from T_X with the p evidence from T_Y, while excluding the p evidence from T_X. Therefore, we need to remove the patient outcomes which happened in T_X after transitioning to p. However, the usual set of evidence we have from T_X is a pair of overall survival (OS) and progression-free survival (PFS) KM curves, introducing a subtle but impactful identification problem.

Call $\{n_s, n_p, n_d\}$ the proportion of patients in $\{s, p, d\}$ in each state, with $n_{\cdot,t}$ being the value at some time t. For simplicity, assume that both KM curves provide values for a common set of time-points $t \in T$. At first glance, the OS and PFS survival curves from T_X should allow us to precisely identify everything we need. The PFS curve provides us with a sequence of $n_{s,t}$, the OS curve provides us with a sequence of $n_{d,t}$, and since all $n_{\cdot,t}$ must add to 1, we can infer a sequence of $n_{p,t}$. Then the intuitive response would be to remove $n_{p,t}$ and replace it with values from T_Y . However, some of the T_X patients in d likely die after being in p, reflecting outcomes

from treatment Z that must also be separated and excluded. Therefore, we can write $n_d = n_{d|s+n_{d|p}}$ as the sum of patients who transitioned $s \rightarrow d$ or $p \rightarrow d$. We need to remove $n_{d|p}$ while keeping $n_{d|s}$, but unfortunately the KM curves (or other published trial evidence) do not provide us with a way to make this separation. Instead, this cleaving of n_d introduces an extra degree of freedom which allows multiple set of parameters to fit the data.

Concretely, take two points in time $t = 1$ and $t = 2$, both in T. We would like to use the T_X survival curves to estimate transition probabilities between states between $t = 1$ and $t = 2$, labeled as $f = \{f_{sp}, f_{sd}, f_{pd}\}$. However, we can only *partially identify* or *set identify* these probabilities over a range of values, since many different combinations of f will fit the observable evidence of $\{n_{s,1}, n_{p,1}, n_{d,1}, n_{s,2}, n_{p,2}, n_{d,2}\}$. To demonstrate this, we bring the following numerically simple example of potential T_X evidence:

Table S6: Example Evidence.

Time	n_s	n_p	n_d
$t = 1$	0.8	0.1	0.1
$t = 2$	0.6	0.2	0.2

We can generate (infinitely) many different combinations of f which fit these data, such as the following values of f' and f^*:

Table S7: Example Probabilities.

	f_{sp}	f_{sd}	f_{pd}
f'	0.25	0	1
f^*	0.1875	0.0625	0.5

Therefore, even if we know $n_{d|s,1}$ and $n_{d|p,1}$ for $t = 1$, f' and f^* both offer different non-refutable possibilities for $n_{d|s,2}$ and $n_{d|p,2}$. Indeed f' shows us that it is possible that no patients transitioned $s \rightarrow d$ while f^* shows us that it is possible that 6.25% of patients did so.

Note that this problem is different from typical statistical issues in pharmacoeconomics or biostatistics. This is not a problem of a misspecified function, such as using Lognormal when the true process follows a Weibull form. This is not an issue of censoring, although censoring potentially introduces a separate identification problem. This is not an issue of sample imprecision, as the example above uses a population-level sample. Finally, this is not an issue of inference in the presence of fixed or random trial-specific effects. The core drive of the identification problem is that the data presented over-aggregrates relevant information: in this case, the history-dependent subgroups $n_{d|s}$ and $n_{d|p}$.

S3.2 Related Literature

We found only a few relevant works that address this identification problem. The first is Li, Litvin, and Manski (2023)[32], who use a global search optimization algorithm to overcome an identification problem in trial reporting, and the second is a 2018 presentation document by Spackman et al.[33], where the presenter outlines our specific identification problem and offers the suggestion of using optimization or simulation approaches. Although neither work offers a comprehensive solution for our needs, we build upon the optimization approach to construct a novel parameter estimation algorithm.

Majer et al. (2022)[29] also appear to approach the problem of estimating three transition parameters from PFS and OS curves, but their solution appears to partly rely on "visual inspection" of goodness-of-fit and does not have a clear discussion of the identification problem, which we found undesirable. Similarly, Pahuta et al. (2019)[30] examine constant transition rates, which is too restrictive for our setting.

Finally, the methodological work of Jansen, Incerti, and Trikalinos (2023)[31] examines a closely related setting. The authors extend the Bayesian network meta-analysis (NMA) approach to use PFS and OS survival curves to jointly estimate pre- and post-progression transition probabilities. Currently, we do not have a solid enough understanding of this method's identifying assumptions (used to overcome the identification problem) to confidently use this

method in our paper. We hope to integrate this extended Bayesian NMA approach in the future as the literature matures.

S3.3 Solution to the Identification Problem

What are some ways to tackle this identification problem? While it may be possible to make credible identifying assumptions to overcome the identification problem entirely and point-identify f, this is an implausible task for all trials in general, especially in the presence of time-varying transition probabilities. Attempting to assume away the identification problem via a general set of assumptions (like a common rate of death between states) would increase the risk of a misspecified model which could behave in unpredictable and poorly understood ways. Alternatively, it is possible to carefully calculate and present the identification sets of transition parameters, and then apply decision-theoretic approaches to rank treatment sequences under uncertainty. However, this approach would be radically different from the existing pharmacoeconomic literature and would require significantly more methodological development first.

Our solution is a middle ground which takes the usual approach of assuming a parametric functional form $f(\theta)$ on f (e.g. usually Weibull or Lognormal form), accepting the partial identification of f by searching over different $f(\theta)$, $\theta \in \Theta$, and finally choosing the $f(\theta^*)$ which maximizes goodness-of-fit. The first major difference in our algorithm is that it jointly estimates all three f values using both PFS and OS survival curves. The usual approach of separately fitting two Lognormal or Weibull functions onto the PFS and OS curves will only estimate the sums $f_{sp}(\theta) + f_{sd}(\theta)$ and $f_{sd}(\theta) + f_{pd}(\theta)$ without additional assumptions, leaving us with uncertainty about the individual values in $f(\theta) = \{f_{sp}(\theta), f_{sd}(\theta), f_{pd}(\theta)\}$ Therefore, we introduce a novel functional form for f that is simple, flexible, and allows for joint estimation of all three transition probabilities. Using our functional form f, our goal is to search across different parameter values $\theta \in \Theta$ and choose the $f(\theta^*)$ which maximizes goodness-of-fit among the broader *set* of possible f that are partially-identified by the OS and PFS curves.

S3.4 Algorithm Overview

We now present an overview of the estimation algorithm for $f(\theta^*)$. We model separate transition probabilities for each combination of treatment and pair of stable/progressed health states. Define the starting health state as s, the progressed health state as p, and being deceased as d. To start, we digitize and store the relevant OS and PFS KM curves. First, we interpolate a "target" OS curve and "target" PFS curve with support along the positive natural numbers up to a maximum corresponding to the inputted KM curves (e.g. 1, 2, . . . 59, 60). Call these target curves C_{os} and C_{pfs} respectively. Call τ_{os} and τ_{pfs} the lengths of C_{os} and C_{pfs}.

Assume time t is discrete and in some range $t \in T = \{1,2,\ldots,\tau\}$. Define τ as the maximum number of periods in the model, with our paper using $\tau = 180$ periods (months) in the base case. Assume that $\tau_{\text{os}}, \tau_{\text{pfs}} \leq \tau$. Define a vector of d parameters $\theta \in \Theta \subset \mathbb{R}^d$and define a numerical grid $\Theta_G \subset \mathbb{R}^d$ that can be computationally searched. Define three transition probabilities $\{f_{sp,t}, f_{sd,t}, f_{pd,t}\}$ as the probability of transitioning $s \to p$ in period t, $s \to d$ in period t, and $p \to d$ in period t, respectively. Now we can define a function f:

$$f\colon \Theta \to [0,1]^{3\tau} \tag{1}$$

where f takes a vector of d parameters $\theta \in \Theta$ outputs a 3 by τ matrix of transition probabilities $f(\theta) = \{(f^{\theta}_{sp,t}, f^{\theta}_{sd,t}, f^{\theta}_{pd,t})\}, t \in$ T where f^{θ} has θ as a superscript and not a power.

Take a continuum of patients n, with $\{n_{s,t}, n_{p,t}, n_{d,t}\}$ defined as the fraction of patients in s, p, or d in period t. Then, we have the following relations describing the true population states in each time period:

$$\begin{aligned} &C_{\text{os}}(t) = n_{s,t} + n_{p,t} && t \in \{1,\ldots,\tau_{\text{os}}\} \\ &C_{\text{pfs}}(t) = n_{s,t} && t \in \{1,\ldots,\tau_{\text{pfs}}\} \quad (2) \\ &n_{s,t} + n_{p,t} + n_{d,t} = 1 && t \in \{1,\ldots,\tau\} \end{aligned}$$

Moreover, given θ, we can simulate a population-level path of patient outcomes by taking starting conditions $\left(n_{s,1}^{\theta}, n_{p,1}^{\theta}, n_{d,1}^{\theta}\right) = (1,0,0)$ and iterating forward to $t = \tau - 1$ using the following relations:

$$\begin{aligned} n_{s,t+1}^{\theta} &= \left(1 - f_{sp,t}^{\theta} - f_{sd,t}^{\theta}\right) n_{s,t}^{\theta} \\ n_{p,t+1}^{\theta} &= \left(1 - f_{pd,t}^{\theta}\right) n_{p,t}^{\theta} + f_{sp,t}^{\theta} n_{s,t}^{\theta} \\ n_{d,t+1}^{\theta} &= n_{d,t}^{\theta} + f_{sd,t}^{\theta} n_{s,t}^{\theta} + f_{pd,t}^{\theta} n_{p,t}^{\theta} \end{aligned} \tag{3}$$

Call these simulated paths $\{n_s^{\theta}, n_p^{\theta}, n_d^{\theta}\}$. Define the error function E as follows:

$$\begin{aligned} E\left(\theta, C_{\text{os}}, C_{\text{pfs}}\right) &= E_{\text{os}} + E_{\text{pfs}} \\ &= \sum_{t=1}^{\tau_{\text{os}}} \left(n_{s,t}^{\theta} + n_{p,t}^{\theta} - C_{\text{os}}(t)\right)^2 + \sum_{t=1}^{\tau_{\text{pfs}}} \left(n_{s,t}^{\theta} - C_{\text{pfs}}(t)\right)^2 \end{aligned} \tag{4}$$

Then choose the parameters θ^* that minimizes this error over Θ^G:

$$\theta^* = \min_{\theta \in \Theta_G} E\left(\theta, C_{\text{os}}, C_{\text{pfs}}\right) \tag{5}$$

Finally, we can use θ^* to construct a set of transition probabilities $\{f(\theta^*, t), t \in T\}$ to simulate patient outcomes in our model.

S3.5 Algorithm Details

KM Curve Interpolation

After digitizing the KM curves and applying the Guyot et al. (2012) algorithm[27] to account for censoring, we need to interpolate the resulting survival curve values over the domains $\{1,2,\dots,\tau_{\text{os}}\}$ and $\{1,2,\dots,\tau_{\text{pfs}}\}$. We use the R *npreg* package to construct smoothing splines, which fit well for all survival curves. The resulting output is the target survival curves C_{os} and C_{pfs} described above. Note that this process is potentially unnecessary if we were to instead specify a continuous-

time process for f. In the end, we chose the discrete-time approach for clarity and simplicity due to the effectiveness of the smoothing spline interpolation and the complication of otherwise introducing an appropriate form of adjusting the error function for different and uneven densities of KM curve observations.

KM Curve Meta-analysis

There is also question of how to incorporate survival curves from different trials for a particular treatment, that is meta-analysis. Typically, a network meta-effects analysis (NMA) approach is used to account for fixed or random trial-specific effects. For example, Jansen, Incerti, and Trikalinos (2023)[29] extend NMA to our setting, namely one with $\{s, p, d\}$ health states where one is trying to estimate $\{f_{sp,t}, f_{sd,t}, f_{pd,t},\ t \in T\}$.

However, we opt not to use their approach because they do not address the identification issue discussed above and it is not entirely clear how the method uses assumptions or Bayesian priors to overcome partial identification along with the consequent effects on estimation bias. Instead of trying to estimate fixed- or random-effects using a partial identification approach, we assume that all KM curves for the same treatment arise from the same data-generating process and fit the smoothing spline to all curves to generate the target survival curves C_{os} and C_{pfs}.

Functional Form of f

The functional form for f needs to be flexible enough to account for different forms of C_{os} and C_{pfs} while being simple enough to avoid over-fitting and having few enough parameters (i.e. a low d) to enable a computational grid-search in a reasonably short time. We settled on having $d = 4$ parameters: p_b representing a "base" probability of f_{sp}, m representing a trend multiplier for p_b, p_{sd} representing constant risk of death f_{sd}, and p_{pd} representing f_{pd}, where f_{sd} is also constant and assumed to be weakly smaller than f_{pd}. Then we have that

$$\theta = \{p_b, m, p_{sd}, p_{pd}\} \tag{6}$$

$$\Theta = [0,1] \times \mathbb{R} \times [0,1] \times [0,1]$$

With this specification for $\theta \in \Theta$, we chose the following functional form for $f(\theta, t)$:

$$\begin{aligned} f &= \{f^{\theta}_{sp,t}, f^{\theta}_{sd,t}, f^{\theta}_{pd,t}\}, \qquad \theta \in \Theta, \qquad t \in T = \{1,2,\dots,\tau\} \\ f^{\theta}_{sp,t} &= p_b(1+m)^{(t-1)} \\ f^{\theta}_{sd,t} &= p_{sd} \\ f^{\theta}_{pd,t} &= p_{pd} \end{aligned} \tag{7}$$

Then we form a grid Θ_G to approximate Θ and computationally search over to find the best fit $f(\theta^*)$ which is then used to simulate treatment sequences.

nmCSPC Modifications

Since there are multiple states to which nmCSPC patients can transition, we needed to modify the $\{s, p, d\}$ structure described above for the nmCSPC health state. First, note that the two available treatments in nmCSPC are intermittent ADT and intermittent enzalutamide + ADT. As shown in Figure 1a, if patients are in active nmCSPC treatment they progress to either nmCRPC or mCRPC, while if they are in suspended treatment they progress to either nmCRPC or mCSPC. However, the published trial evidence[2] only provides an OS KM survival curve and a single "metastasis-free survival" KM curve.

Therefore, we keep the $\{s, p, d\}$ structure using either active or suspended treatment in nmCSPC as the health state s, with transitions between these two states described in Section 2 of the appendix. Aligning with the KM survival curve data, p is a progression to metastases, either mCRPC from active treatment or mCSPC from suspended treatment.

Concerning evidence on patients' transitions to nmCRPC, we have a total event number for the "castration resistance" event but no corresponding KM survival curve. Moreover, we do not have any information on the joint incidence of metastasis and castration resistance (i.e. no event recording progression to mCRPC). Given the sparsity of data, we make additional assumptions about the nmCSPC to nmCRPC transition to constrain our estimation. First, we assume that metastasis and castration resistance are independent, that is the rate of transition from nmCSPC to mCSPC is the same as the rate of transition from nmCRPC to mCRPC. Second, we

assume that the ratio of m to p_b is the same for metastasis as castration resistance, so both transition probabilities follow a similar trend over time. We then estimate nmCSPC to nmCRPC transition probabilities using a similar $\{s, p\}$ method as for metastasis, except the error is calculated against a single total event value (at time τ_{pfs}) and not a sequence of values C_{pfs}.

Finally, we need to estimate the proportion of nmCSPC to mCSPC patients who enter low-risk versus high-risk mCSPC. We use the estimate from Francini et al. (2018)[1] of 65.1% of mCSPC patients (post-nmCSPC) entering low-risk mCSPC.

S4 Transition Probability Parameter Estimates

S4.1 Estimated Parameter Values

Table S8: List of transition probability parameter values.

Starting State	Treatment	p_b	m	p_{sd}	p_{pd}
low-risk mCSPC	adt	0.0168	0.0000	0.0003	0.0334
	abi	0.0042	0.0083	0.0011	0.1143
	apa	0.0126	-0.0083	0.0010	0.0958
	doce+abi	0.0043	0.0417	0.0017	0.0334
	daro	0.0132	0.0020	0.0010	0.1025
	doce+daro	0.0123	0.0000	0.0008	0.0776
	doce+enza	0.0043	0.0417	0.0013	0.0251
	enza	0.0043	0.0083	0.0006	0.0584
high-risk mCSPC	adt	0.0417	-0.0167	0.0050	0.0376
	abi	0.0184	-0.0083	0.0008	0.0817
	apa	0.0126	-0.0083	0.0010	0.0958
	daro	0.0132	0.0020	0.0010	0.1025
	doce	0.0251	0.0000	0.0062	0.0209
	doce+abi	0.0126	0.0083	0.0023	0.0459
	doce+daro	0.0126	0.0000	0.0008	0.0750
	doce+enza	0.0126	0.0000	0.0034	0.0376
	enza	0.0126	-0.0292	0.0094	0.0126
nmCRPC	adt	0.0388	0.0020	0.0002	0.0184
	abi	0.0001	0.0500	0.0021	0.0417
	apa	0.0084	0.0417	0.0003	0.0334
	daro	0.0126	0.0000	0.0019	0.0209
	enza	0.0084	0.0417	0.0005	0.0459
nmCSPC (to mCSPC/mCRPC)	intermittent adt	0.0032	0.0143	0.0008	0.0154
	intermittent enza	0.0021	0.0020	0.0002	0.0245
nmCSPC (to nmCRPC)	intermittent adt	0.0035	0.0157	0.0008	0.0154
	intermittent enza	0.0004	0.0004	0.0020	0.0245
mCRPC	abi	0.0376	0.0083	0.0005	0.0542
	enza	0.0251	0.0250	0.0006	0.0625
	doce	0.0959	0.0667	0.0064	0.0368
progressed mCRPC	abi (post doce)	0.0695	0.0667	0.0163	0.0939
	caba (post doce)	0.1796	0.0347	0.0035	0.0695
	enza (post doce)	0.0459	0.0667	0.0161	0.0750

S4.2 Comparison of Estimation Methods

Our parameter estimation method fits the survival curves similarly well compared to the standard approaches of fitting KM survival curves using Lognormal or Weibull forms. This is despite the fact that our method is directly estimating parameters for the three state-transition probabilities in our setting (as in Table S3.2). By contrast, the standard methods estimate patient populations in each health state (as in Table S3.1), which would still require additional assumptions or another estimation step to address the underlying identification problem for the state-transition probabilities $\{f_{sp,t}, f_{sd,t}, f_{pd,t}\}$.

The following table shows the relative goodness-of-fit of each method by comparing the sum of mean-squared errors for the PFS and OS KM survival curves (i.e. $E(\theta, C_{\text{os}}, C_{\text{pfs}})$) for our novel estimation method versus standard methods of fitting using Lognormal and Weibull functions. We see that although our method does not typically deliver the lowest MSE, it performs comparably well to the minimum MSE choice between a Lognormal and Weibull fit across all treatments.

Also, we visually demonstrate the goodness-of-fit of each method for our inputted KM survival curves in the figures below. Again, we demonstrate the goodness-of-fit and well-behaved extrapolations of the KM curves generated by our estimated parameters.

Table S9: MSE of different parameter estimation approaches.

Starting State	Treatment	Novel Method	Lognormal	Weibull
low-risk mCSPC	adt	0.0058	0.0088	0.0374
	abi	0.0064	0.0006	0.0107
	apa	0.0066	0.0017	0.0536
	doce+abi	0.0285	0.0093	0.0083
	daro	0.0051	0.0007	0.0156
	doce+daro	0.0230	0.0057	0.1112
	doce+enza	0.0113	0.0033	0.0055
	enza	0.0131	0.0064	0.0108
high-risk mCSPC	adt	0.0177	0.0107	0.1321
	abi	0.0339	0.0136	0.2180
	apa	0.0066	0.0015	0.0495
	daro	0.0051	0.0007	0.0156
	doce	0.0390	0.0038	0.1231
	doce+abi	0.0259	0.0052	0.0695
	doce+daro	0.0160	0.0056	0.0564
	doce+enza	0.0409	0.0104	0.0565
	enza	0.0069	0.0020	0.0090
nmCRPC	adt	0.0066	0.0057	0.1594
	abi	0.0049	0.0016	0.0031
	apa	0.0030	0.0033	0.0133
	daro	0.0042	0.0105	0.0186
	enza	0.0043	0.0042	0.0147
nmCSPC	intermittent adt	0.0274	0.0075	0.0779
	intermittent enza	0.0037	0.0018	0.0066
mCRPC	abi	0.0218	0.0061	0.0370
	doce	0.0140	0.0095	0.0182
	enza	0.0107	0.0090	0.0329
progressed mCRPC	abi (post doce)	0.0272	0.0194	0.0735
	caba (post doce)	0.0103	0.0474	0.0078
	enza (post doce)	0.0135	0.0189	0.0269

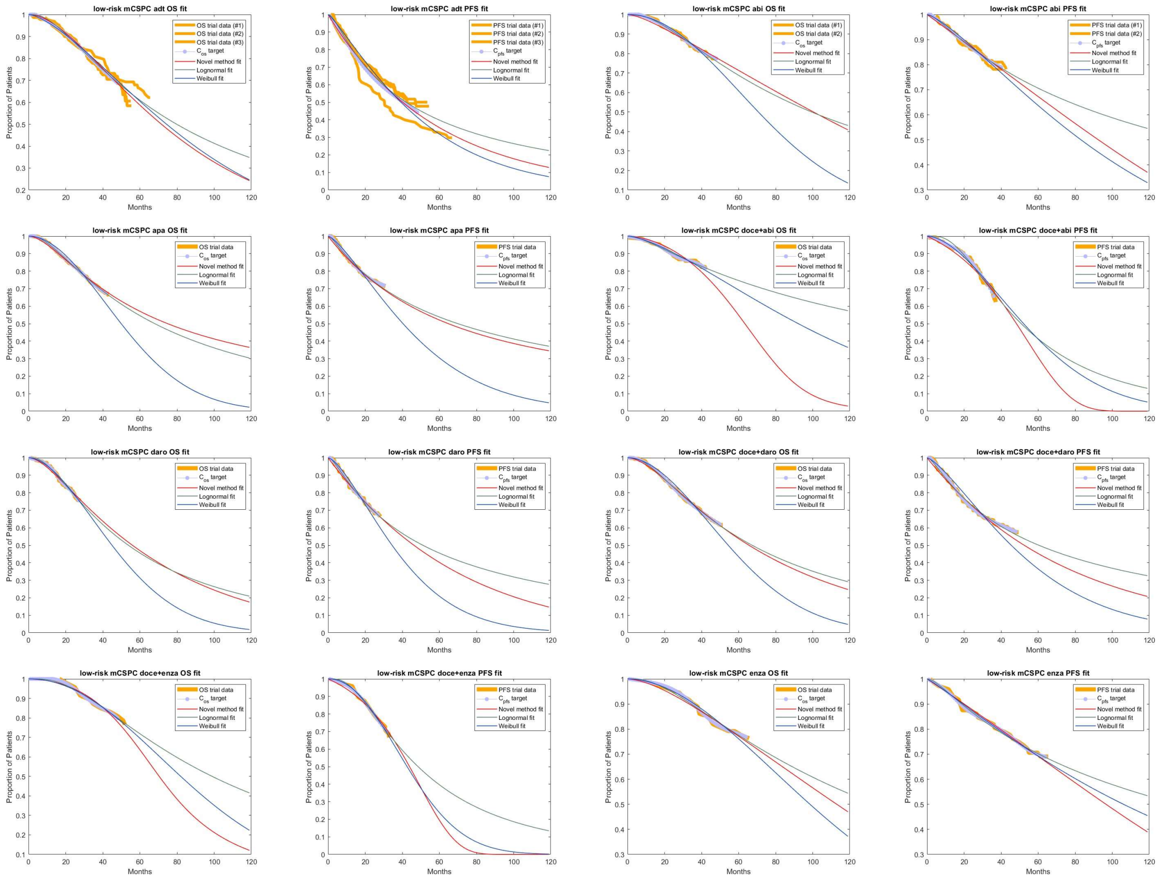

Figure S1: KM curve fitting comparisons for low-risk mCSPC.

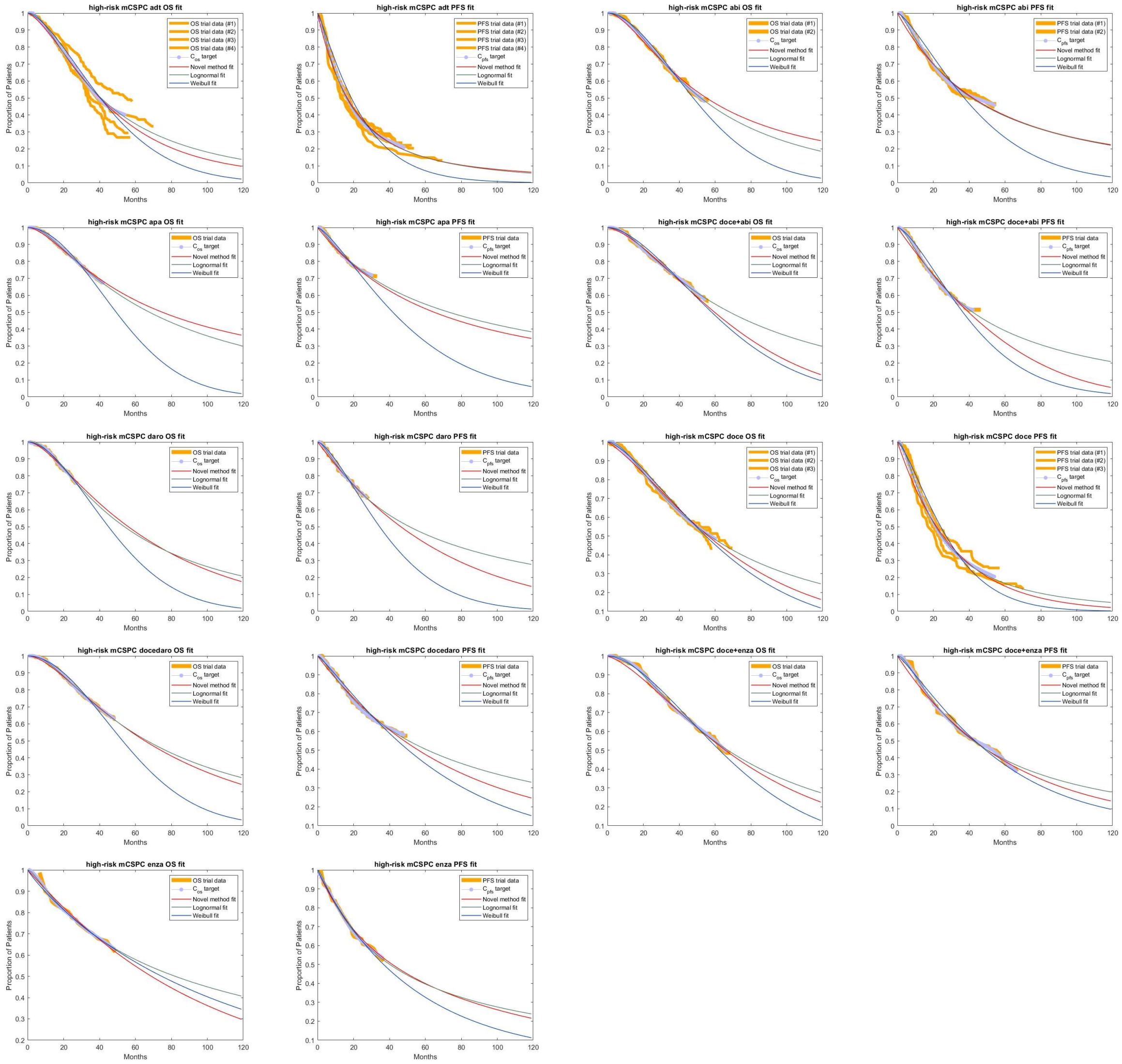

Figure S2: KM curve fitting comparisons for high-risk mCSPC.

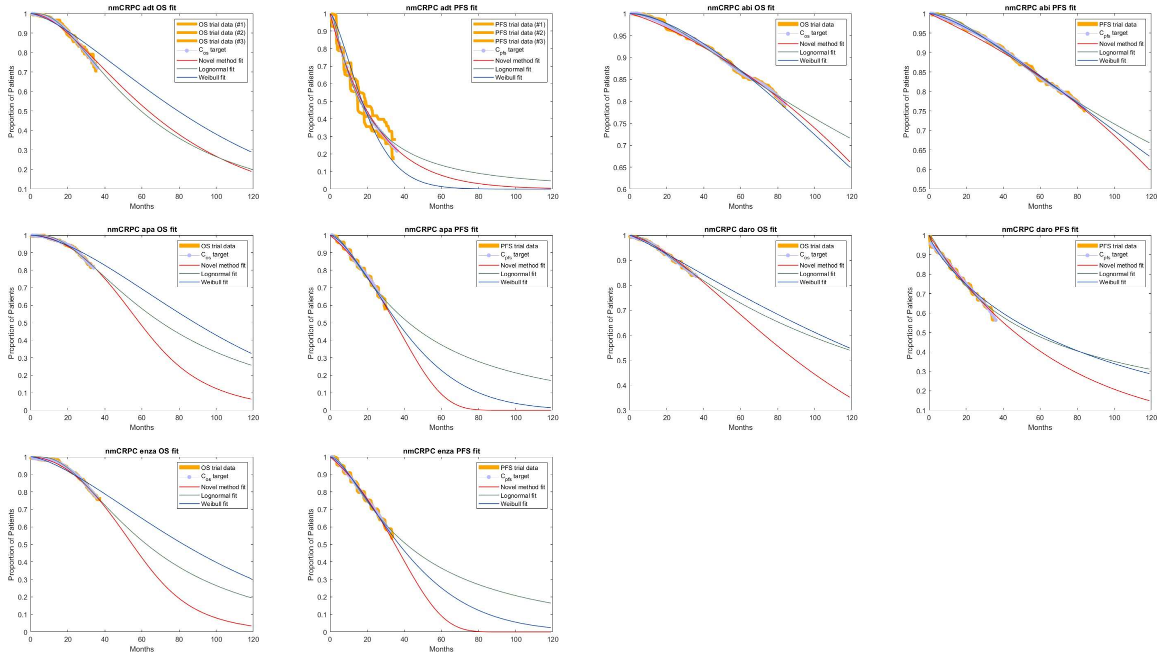

Figure S3: KM curve fitting comparisons for nmCRPC.

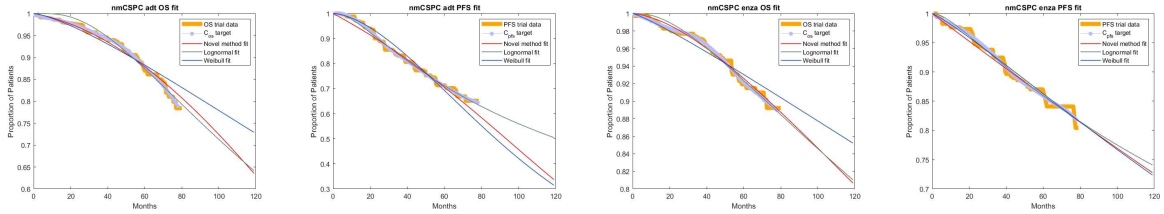

Figure S4: KM curve fitting comparisons for nmCSPC.

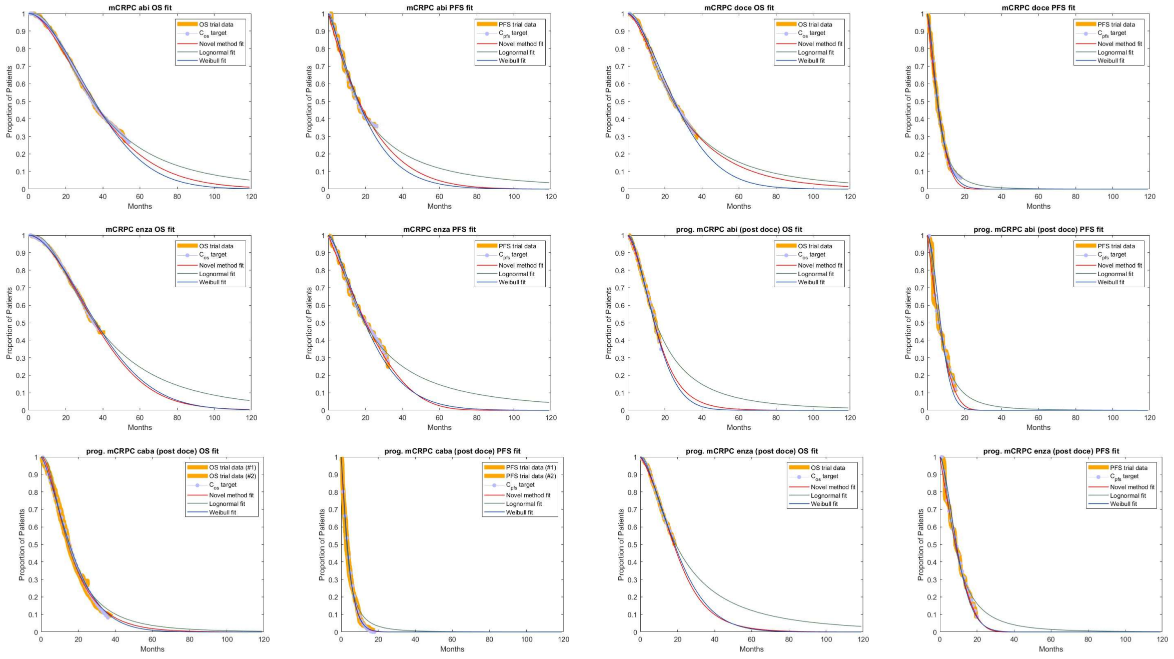

Figure S5: KM curve fitting comparisons for mCRPC.

S5 Additional Figures and Tables

S5.1 Acceptability Curve Graphs: Individual Sequences

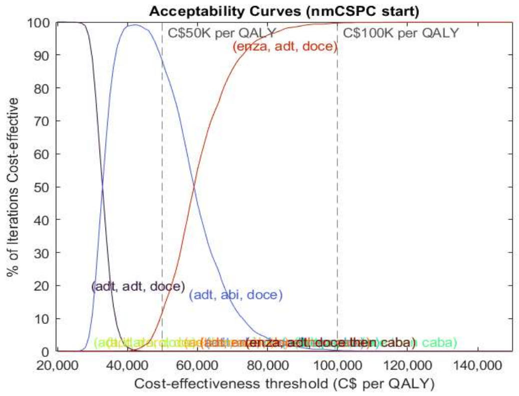

a) nmCSPC start

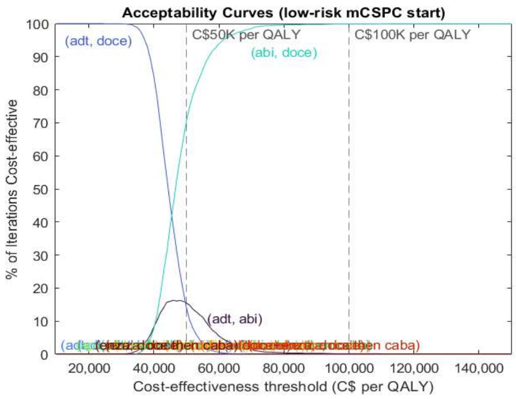

b) low-risk mCSPC start

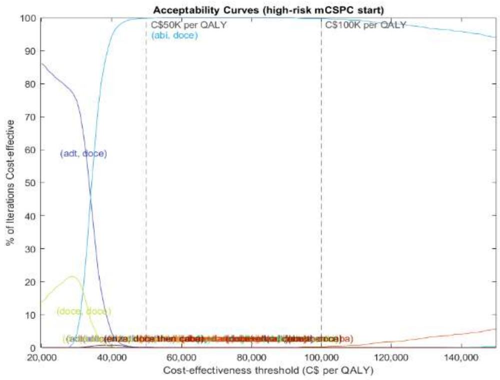

c) high-risk mCSPC start

Figure S6: Cost-effectiveness acceptability curves: individual sequences.

S5.2 Benefit-Cost Plots: Individual Sequences

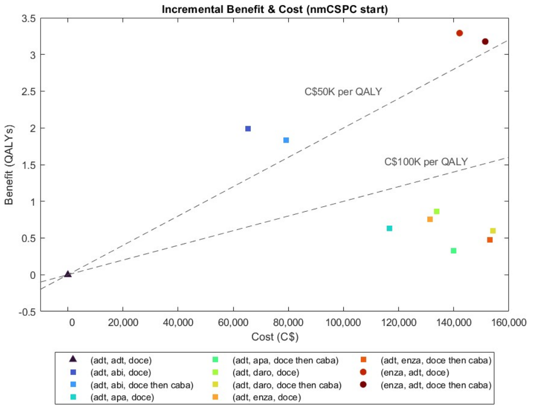

a) nmCSPC start

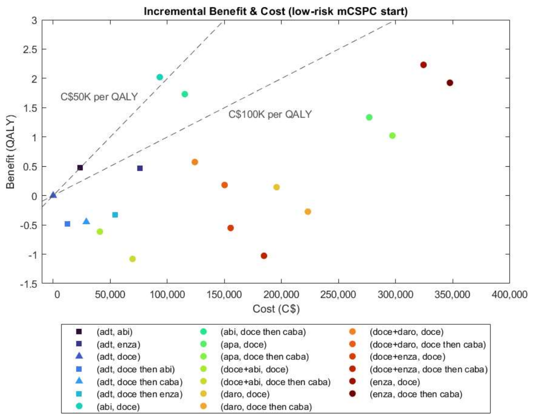

b) low-risk mCSPC start

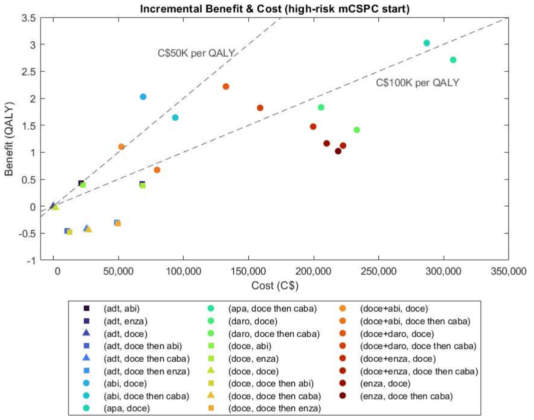

c) high-risk mCSPC start

Figure S7: Benefit-cost plots: individual sequences.

S5.3 Benefit-Cost Plots: Grouped Sequences

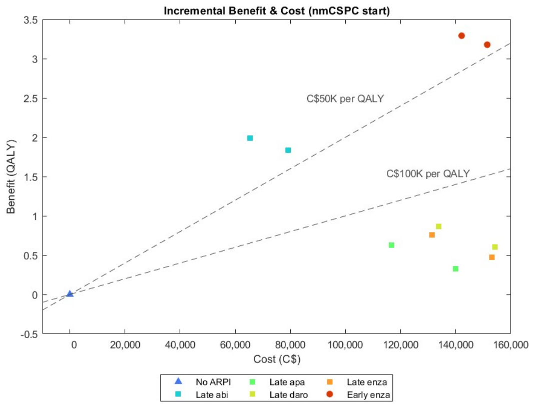

a) nmCSPC start

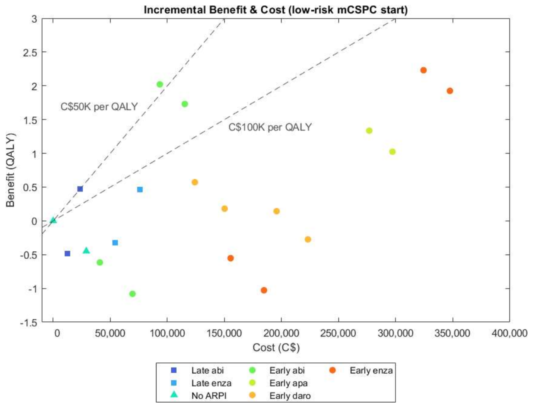

b) low-risk mCSPC start

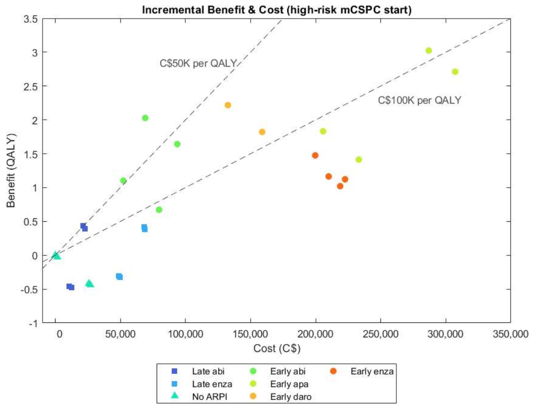

c) high-risk mCSPC start

Figure S8: Benefit-cost plots: grouped sequences.

S5.4 Acceptability Curve Graphs: Grouped Sequence Pairwise Comparisons vs. no ARPI

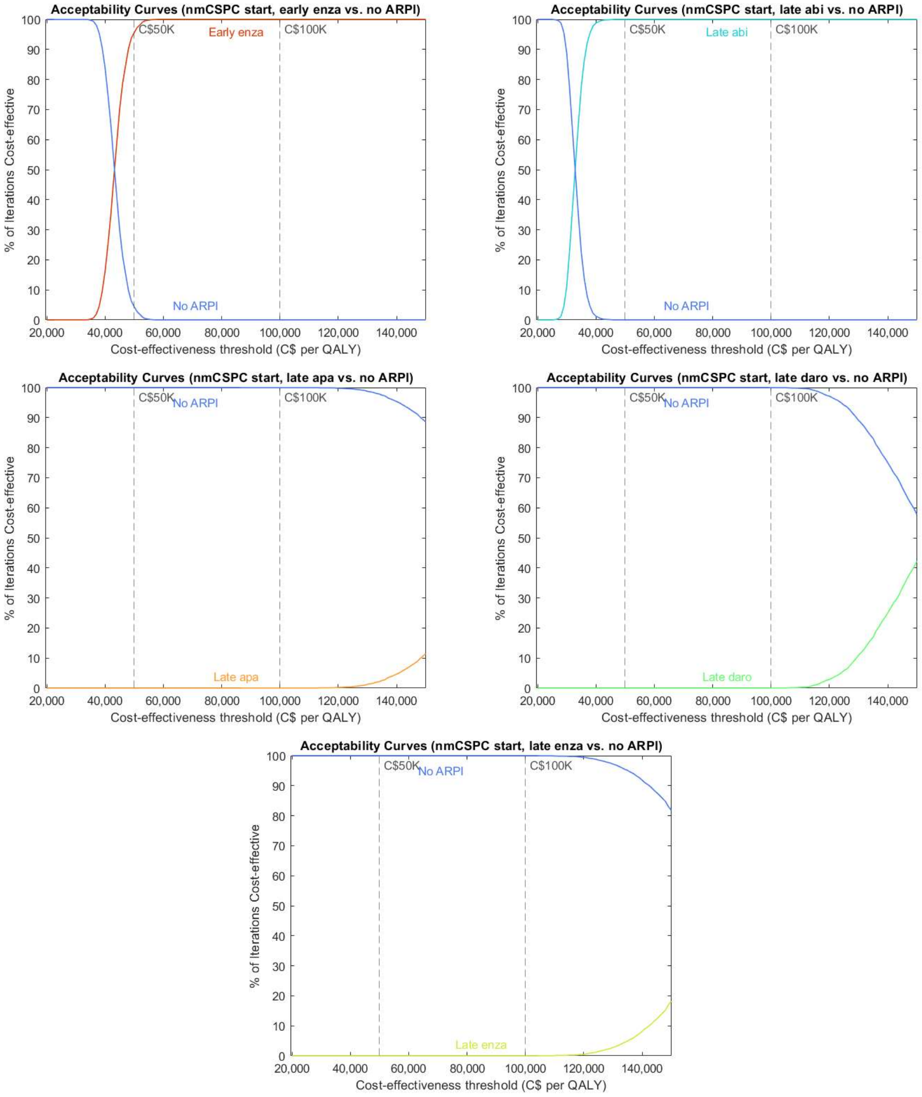

Figure S9: Pairwise Comparisons of Grouped Sequences: nmCSPC Start.

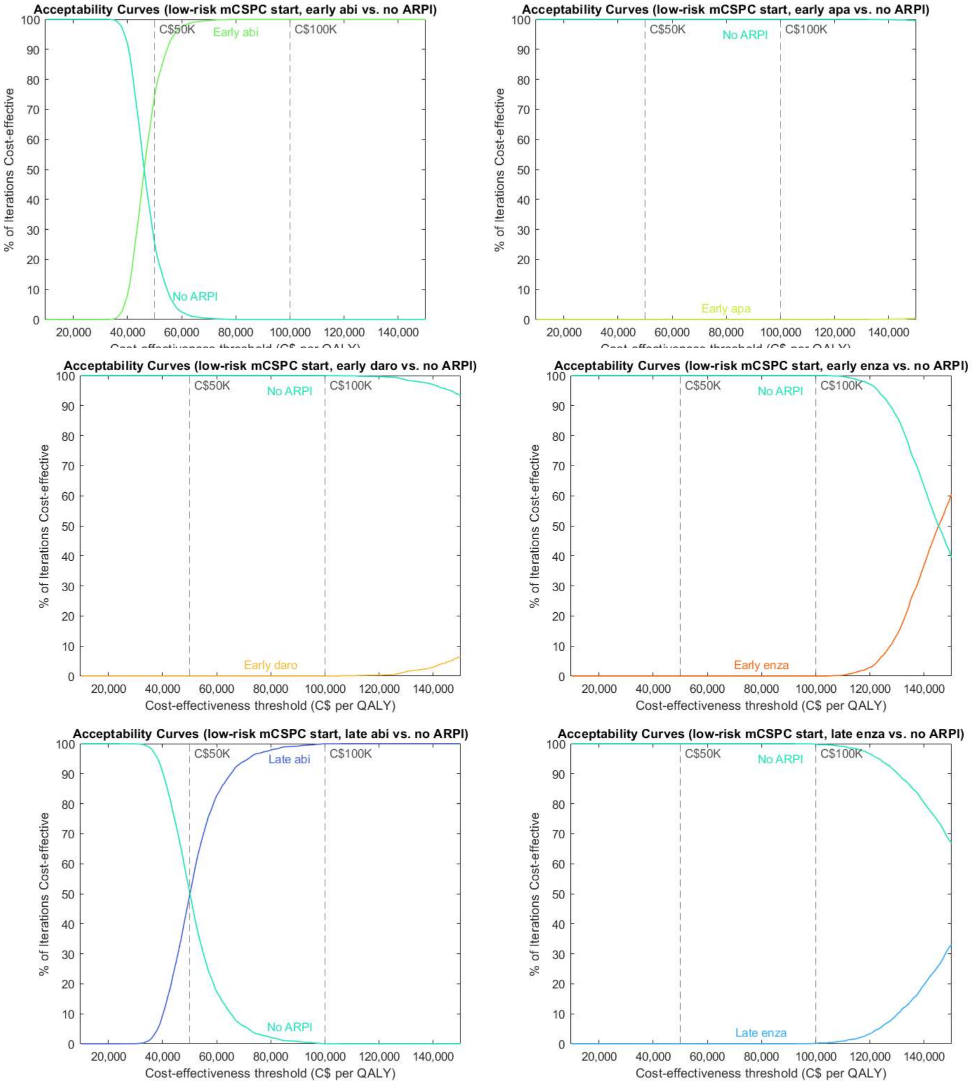

Figure S10: Pairwise Comparisons of Grouped Sequences: Low-risk mCSPC Start.

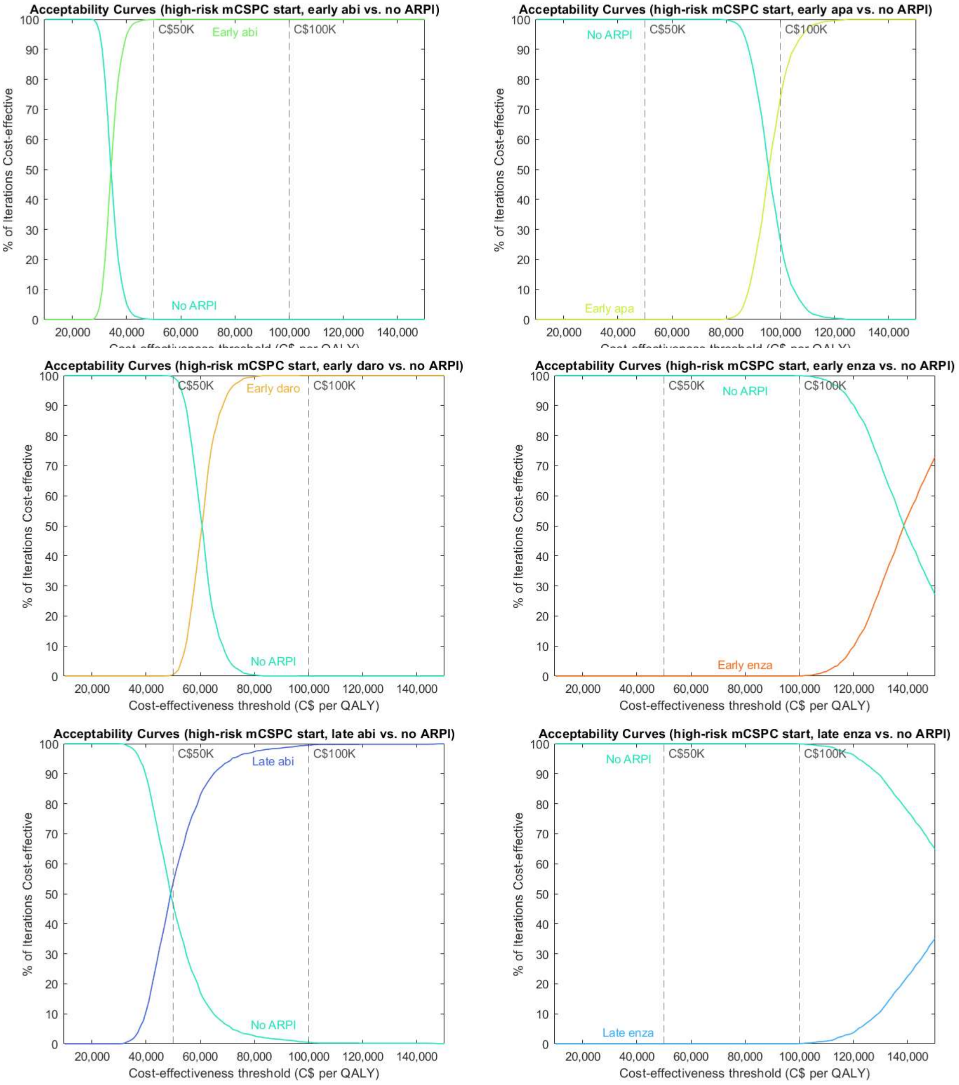

Figure S11: Pairwise Comparisons of Grouped Sequences: High-risk mCSPC Start.

S5.5 Selected Cumulative Distribution Function (CDF) Graphs of Total NHB

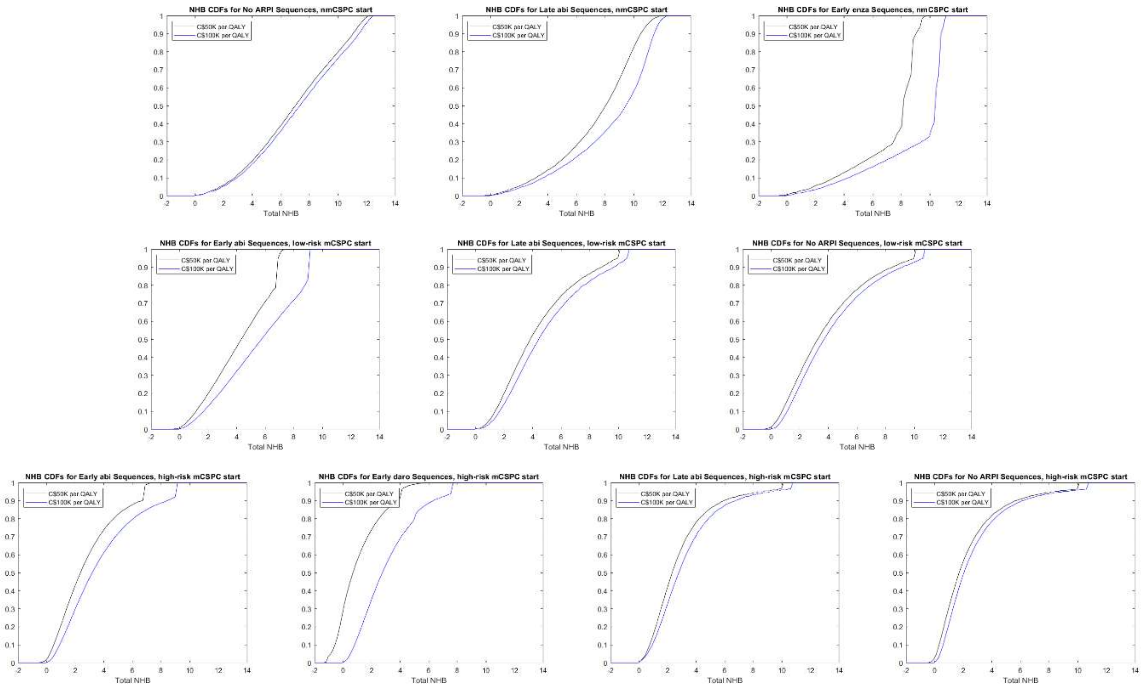

Figure S12: Selected CDF Graphs of Total NHB for Grouped Sequences.

S5.6 Time-horizon Sensitivity Analysis Tables

Table S10: Most cost-effective sequences with a 10-year time horizon.

Starting Health State	Cost Effectiveness Threshold	Rank	Incremental NHB (QALY)	Total NHB (QALY)	Treatment Sequence (nmCSPC, nmCRPC/mCSPC, mCRPC)* or (mCSPC, mCRPC)†	ARPI Use
nmCSPC	C$50K per QALY	1	0.10	6.26	(adt, abi, doce)*	late
		2	0.00	6.17	(adt, adt, doce)*	none
		3	-0.10	6.06	(adt, abi, doce then caba)*	late
	C$100K per QALY	1	0.48	6.89	(adt, abi, doce)*	late
		2	0.35	6.76	(adt, abi, doce then caba)*	late
		3	0.29	6.70	(enza, adt, doce)*	early
low-risk mCSPC	C$50K per QALY	1	0	4.03	(adt, doce)†	none
		2	-0.01	4.02	(adt, abi)†	late
		3	-0.23	3.80	(abi, doce)†	early
	C$100K per QALY	1	0.54	4.87	(abi, doce)†	early
		2	0.21	4.54	(abi, doce then caba)†	early
		3	0.20	4.53	(adt, abi)†	late
high-risk mCSPC	C$50K per QALY	1	0.33	3.09	(abi, doce)†	early
		2	0.06	2.82	(doce+abi, doce)†	early
		3	0.03	2.80	(doce, abi)†	late
	C$100K per QALY	1	0.90	3.88	(abi, doce)†	early
		2	0.57	3.54	(doce+abi, doce)†	early
		3	0.56	3.54	(doce+daro, doce)†	early

Table S11: Most cost-effective sequences with a 20-year time horizon.

Starting Health State	Cost Effectiveness Threshold	Rank	Incremental NHB (QALY)	Total NHB (QALY)	Treatment Sequence (nmCSPC, nmCRPC/mCSPC, mCRPC)* or (mCSPC, mCRPC)†	ARPI Use
nmCSPC	C$50K per QALY	1	1.81	8.97	(enza, adt, doce)*	early
		2	1.40	8.56	(enza, adt, doce then caba)*	early
		3	1.16	8.32	(adt, abi, doce)*	late
	C$100K per QALY	1	3.52	11.00	(enza, adt, doce)*	early
		2	3.23	10.71	(enza, adt, doce then caba)*	early
		3	1.95	9.43	(adt, abi, doce)*	late
low-risk mCSPC	C$50K per QALY	1	0.34	4.91	(abi, doce)†	early
		2	0.01	4.58	(adt, abi)†	late
		3	0	4.57	(adt, doce)†	none
	C$100K per QALY	1	1.33	6.25	(abi, doce)†	early
		2	0.71	5.63	(abi, doce then caba)†	early
		3	0.25	5.17	(adt, abi)†	late
high-risk mCSPC	C$50K per QALY	1	0.85	3.90	(abi, doce)†	early
		2	0.01	3.06	(adt, abi)†	late
		3	0	3.05	(adt, doce)†	none
	C$100K per QALY	1	1.62	4.91	(abi, doce)†	early
		2	1.00	4.29	(doce+daro, doce)†	early
		3	0.96	4.25	(abi, doce then caba)†	early

S5.7 Price Sensitivity Analysis Table

Table S12: Most cost-effective sequences with equal ARPI prices.

Starting Health State	Cost Effectiveness Threshold	Rank	Incremental NHB (QALY)	Total NHB (QALY)	Treatment Sequence (nmCSPC, nmCRPC/mCSPC, mCRPC)* or (mCSPC, mCRPC)†	ARPI Use
nmCSPC	C$50K per QALY	1	2.51	9.47	(enza, adt, doce)*	early
		2	2.20	9.17	(enza, adt, doce then caba)*	early
		3	0.68	7.65	(adt, abi, doce)*	late
	C$100K per QALY	1	2.90	10.17	(enza, adt, doce)*	early
		2	2.69	9.96	(enza, adt, doce then caba)*	early
		3	1.34	8.61	(adt, abi, doce)*	late
low-risk mCSPC	C$50K per QALY	1	0.29	4.73	(enza, doce)†	early
		2	0.15	4.59	(abi, doce)†	early
		3	0	4.44	(adt, doce)†	none
	C$100K per QALY	1	1.26	6.03	(enza, doce)†	early
		2	1.09	5.86	(abi, doce)†	early
		3	0.72	5.50	(enza, doce then caba)†	early
high-risk mCSPC	C$50K per QALY	1	1.27	4.22	(doce+daro, doce)†	early
		2	1.22	4.17	(apa, doce)†	early
		3	0.65	3.60	(abi, doce)†	early
	C$100K per QALY	1	2.12	5.31	(apa, doce)†	early
		2	1.74	4.93	(doce+daro, doce)†	early
		3	1.61	4.79	(apa, doce then caba)†	early

S5.8 Alternate Payer Costs Table

Table S13: Alternate payer costs.

Treatment	US Public Payer Cost (US$, monthly)	US Private Payer Cost (US$, monthly)	UK Public Payer Cost (£, monthly)
ADT	154	542.03	70
Abiraterone	405.78	6035.40	2745
Apalutamide	11350	16231	2735
Darolutamide	10447	14704	4040
Docetaxel	135.01	510.97	101
Enzalutamide	7909	15600	2735

References

2. Freedland SJ, Luz MdA., De Giorgi U et al. Improved Outcomes with Enzalutamide in Biochemically Recurrent Prostate Cancer. *New England Journal of Medicine*. 2023;389(16):1453–1465.

27. Guyot P, Ades A, Ouwens MJ, and Welton NJ. Enhanced secondary analysis of survival data: re- constructing the data from published Kaplan-Meier survival curves. *BMC Medical Research Methodology*. 2012;12(1):9.

29. Majer I., Kroep S., Maroun R., Williams C., Klijn S., and Palmer S. Estimating and Extrapolating Survival Using a State-Transition Modeling Approach: A Practical Application in Multiple Myeloma. Value in Health. 2022;25(4):595–604.

30. Pahuta M.A., Werier J., Wai E.K., Patchell R.A., and Coyle D. A technique for approximating transition rates from published survival analyses. Cost Effectiveness and Resource Allocation. 2019;17(1):12.

31. Jansen J.P., Incerti D., and Trikalinos T.A. Multi-state network meta-analysis of progression and survival data. Statistics in Medicine. 2023;42(19):3371–3391.

32. Li S., Litvin V., and Manski C.F.. Partial Identification of Personalized Treatment Response with Trial-reported Analyses of Binary Subgroups. Epidemiology. 2023;34(3):319.

33. Spackman E., Weaver C., Danthurebandara V., Faria R., and Soares, M. Estimating Markov State Transition Probabilities from Published Kaplan-Meier Survival Curves Presented at CADTH Symposium 2018; April 16 2018. https://www.cda-amc.ca/sites/default/files/symp-2018/presentations/april16-2018/Concurrent-Session-B8-Eldon-Spackman.pdf

45. Francini E, Gray KP, Xie W, et al. Time of metastatic disease presentation and volume of disease are prognostic for metastatic hormone sensitive prostate cancer (mHSPC). *The Prostate.* 2018;78(12):889–895.
